# Extracellular vesicles in solid tumors: from tumor ecology to engineered therapeutics

**DOI:** 10.1186/s12943-026-02705-7

**Published:** 2026-06-20

**Authors:** Wenxun Cai, Yuhang Chen, Suoyi Dai, Chien-Shan Cheng, Lianyu Chen

**Affiliations:** 1 Department of Integrative Oncology, Fudan University Shanghai Cancer Center, Shanghai, 200032 China; 2 Department of Oncology, Shanghai Medical College, Fudan University, 270 Dong-An Road, Shanghai, 200032 China

**Keywords:** Extracellular vesicles, Solid tumors, Tumor microenvironment, Metastasis, Liquid biopsy, Drug delivery, Cancer immunotherapy, Therapy resistance, EV engineering, Translational challenges

## Abstract

Extracellular vesicles (EVs) are important mediators of intercellular communication in solid tumors. Released by malignant, stromal, immune, and microbial cells, they influence tumor evolution by transferring proteins, nucleic acids, lipids, and metabolites that reshape local and systemic signaling. Current evidence implicates EVs in tumor microenvironment remodeling, metastatic niche formation, immune regulation, and adaptive responses to metabolic and therapeutic stress. However, these functions are highly context-dependent and remain unevenly supported across tumor types, disease stages, and experimental systems. Mechanistically, EV production is increasingly understood not as a constitutive secretory event, but as an adaptive output of intracellular trafficking and metabolic programs that govern vesicle fate, cargo selection, and release under stress. The same properties that complicate biological interpretation—including heterogeneity, membrane plasticity, and context-dependent cargo sorting—also make EVs attractive candidates for therapeutic engineering. In this Review, we critically examine EV biology in solid tumors by connecting biogenesis, trafficking control, lipid metabolism, and functional heterogeneity with emerging engineering strategies, including source selection, surface modification, cargo loading, and hybrid engineering strategies. We further discuss the major barriers that continue to limit clinical translation, particularly biological heterogeneity, isolation-dependent variability, incomplete mechanistic resolution, manufacturing scalability, and regulatory standardization. By distinguishing more established principles from emerging or model-restricted findings, this Review aims to provide a balanced assessment of both the opportunities and the current limitations of EV-based diagnostics and therapeutics.

## Introduction

Extracellular vesicles (EVs) are lipid bilayer-enclosed particles released by virtually all cell types and are now widely regarded as important components of intercellular communication. In solid tumors, EVs operate within a broader signaling landscape that also includes soluble factors, metabolites, and direct cell–cell interactions. Rather than acting as isolated or universally dominant drivers, EVs are increasingly understood as context-dependent communication nodes that link tumor-intrinsic programs with stromal, immune, vascular, and metabolic responses across local and systemic scales. Through the transfer of selected proteins, nucleic acids, lipids, and other bioactive cargoes, EVs have been implicated in tumor microenvironment remodeling, metastatic dissemination, immune regulation, and therapeutic adaptation [1]. At the same time, these same biological features have stimulated growing interest in EVs as engineering substrates for drug delivery and immunomodulation. This dual identity—as both mediators of tumor-associated signaling and candidate therapeutic platforms—has made EV biology an increasingly prominent area of cancer research.

EVs comprise a heterogeneous and operationally defined population of vesicles that are commonly classified according to biogenesis, size, molecular composition, and physical properties. The major subtypes include exosomes, which arise from the endosomal system, and microvesicles (also referred to as ectosomes or microparticles), which are generated by outward budding of the plasma membrane [2–4]. In practice, however, these categories are not always readily separable, because vesicles of distinct biogenetic origin often overlap in size, density, and marker distribution, and most currently used isolation methods rely precisely on these shared physicochemical features [2]. To improve rigor and comparability, the International Society for Extracellular Vesicles (ISEV) recommends the use of the broader term “extracellular vesicles” (EVs), together with operational subclasses, when biogenesis cannot be assigned with confidence [2]. In accordance with this framework, we use “EVs” throughout this Review unless a cited study explicitly defines a vesicle subtype on the basis of biogenesis or subtype-specific nomenclature.

Beyond their roles in tumor-associated communication, EVs also possess properties that support their development as therapeutic platforms, including membrane flexibility, biocompatibility, relative protection of molecular cargo, and the capacity to interact with biological barriers and recipient cells [5]. At the same time, EV biology in cancer is increasingly being understood in the context of intracellular trafficking and metabolic programs that shape vesicle formation, cargo selection, and release. Endosomal sorting, endosome–lysosome balance, secretory autophagy, recycling pathways, and lipid metabolic rewiring are emerging as interconnected determinants of EV output, particularly under tumor-associated stress conditions. These mechanistic advances have begun to inform EV engineering aimed at improving cargo loading, targeting specificity, pharmacokinetics, and delivery performance.

Despite growing interest in EV-based diagnostics and therapeutics, biological heterogeneity, isolation-dependent variability, incomplete mechanistic resolution, and unresolved questions of generalizability continue to limit interpretation and translation. These constraints also shape the critical perspective of this Review. In this Review, we examine EVs in solid tumors from both biological and translational perspectives, with particular emphasis on how advances in EV biogenesis, trafficking control, lipid metabolism, and multicellular signaling are informing engineering strategies for diagnostics and therapeutics. We distinguish more established concepts from emerging or model-dependent observations to provide a critical framework for understanding both the opportunities and current limitations of EV-based approaches in precision oncology.

## EV Biology: structure, biogenesis, and functional diversity

### Structure and composition of EVs

EVs are anucleated membrane-bound particles that range from nano- to micro-scale and carry diverse biomolecules derived from their parent cells. Despite marked heterogeneity in size, origin, and molecular composition, EVs share a common structural logic: a lipid bilayer membrane associated with selectively incorporated proteins, nucleic acids, lipids, and metabolites that together support intercellular communication. Among the principal subclasses, exosomes and microvesicles are traditionally distinguished by their routes of biogenesis and related structural properties. However, these categories are not always experimentally separable, because vesicles of different origin frequently overlap in size, density, and molecular markers. For this reason, EV subtypes are often better interpreted operationally and along a continuum rather than as strictly discrete entities [6, 7].

Exosomes arise from the endosomal system as intraluminal vesicles (ILVs) within multivesicular bodies (MVBs) and are released when MVBs fuse with the plasma membrane, whereas microvesicles bud directly from the plasma membrane [1, 8]. This distinction broadly aligns with differences in size, with exosomes typically measuring 30–150 nm and microvesicles approximately 100–1,000 nm, although substantial overlap exists [8, 9]. Their formation also depends on partly distinct molecular mechanisms: exosome biogenesis commonly involves ESCRT-dependent and lipid-mediated pathways, whereas microvesicle shedding is more closely associated with plasma membrane remodeling, cytoskeletal rearrangement, and calcium signaling [10]. These routes are accompanied by differences in membrane composition. Exosome membranes are often enriched in cholesterol, sphingomyelin, and ceramide, whereas microvesicle membranes more closely resemble the parent plasma membrane [11]. Beyond structural roles, current evidence indicates that cholesterol, sphingolipids, ceramide, and related lipid species can influence membrane curvature, vesicle budding, cargo sorting, and interactions with recipient cells, suggesting that the EV membrane acts as an active molecular interface rather than a passive shell [6, 12, 13].

The molecular cargo of EVs underlies their biological activity. Transmembrane proteins such as the tetraspanins CD9, CD63, and CD81 contribute to vesicle organization, recognition, and trafficking, while cytosolic and luminal proteins—including RAB GTPases, ESCRT-associated factors, heat shock proteins, and metabolic enzymes—support vesicle biogenesis and intercellular signaling. EVs also carry diverse nucleic acids, including mRNA, miRNA, lncRNA, and DNA fragments [1, 14, 15]. Once transferred to recipient cells, these cargoes can alter transcriptional programs and cellular phenotypes [1, 10, 11]. Importantly, EV cargo composition is not simply a passive reflection of donor-cell contents, but a dynamic output shaped by intracellular sorting, trafficking state, and metabolic context. In cancer, this regulated cargo selection likely contributes to EV functional heterogeneity and is closely linked to the trafficking programs discussed below [6, 16].

### EV Biogenesis as an intracellular trafficking network

**Fig. 1:**
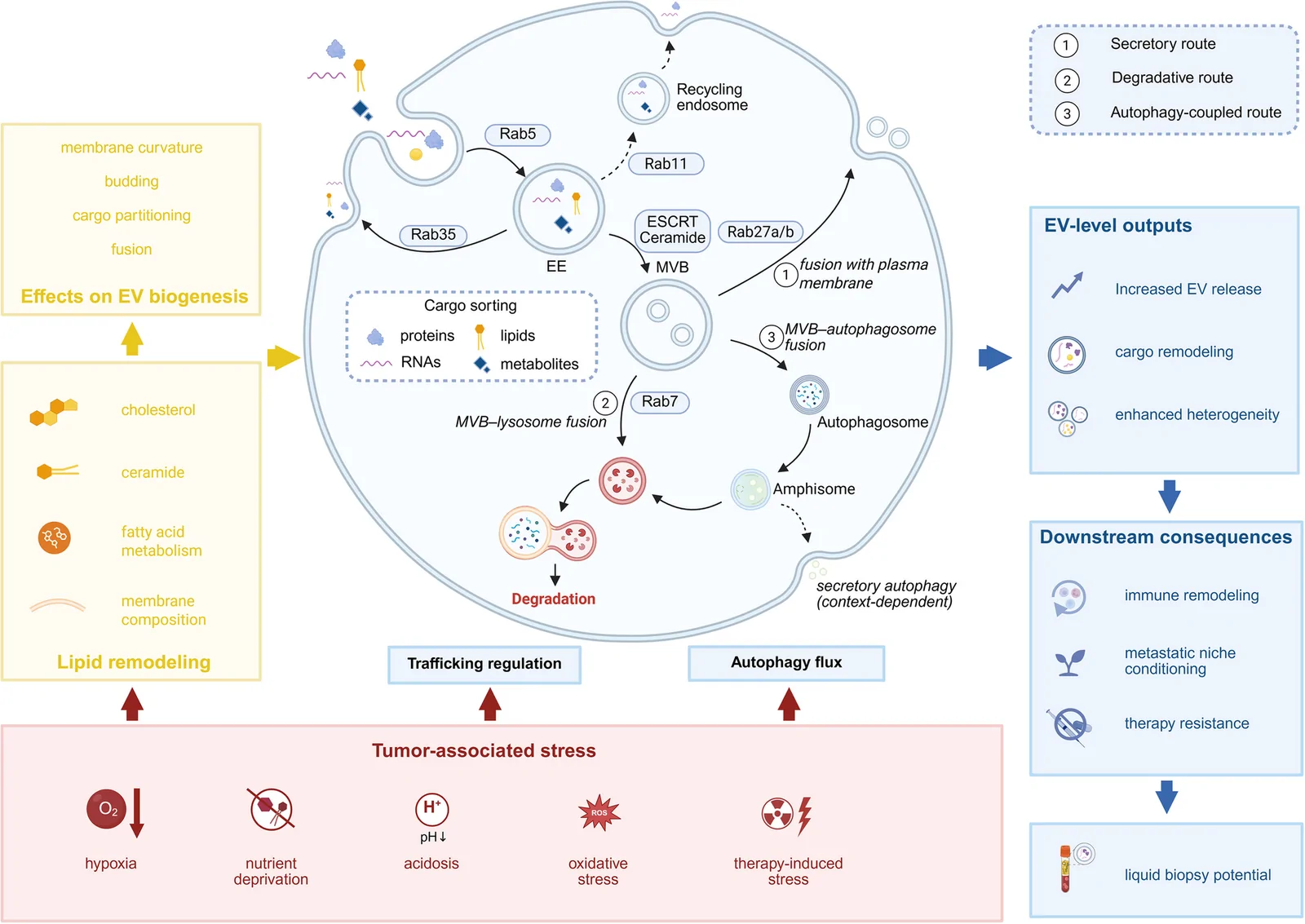
Integrated control of EV biogenesis by intracellular trafficking, autophagy, and lipid remodeling in solid tumors. This schematic illustrates EV biogenesis as an adaptive output of intracellular trafficking and metabolic control in tumor cells. Following endocytosis, cargo is selectively sorted through the endosomal system into MVBs, which function as a trafficking decision hub with three major fates: (1) fusion with the plasma membrane for EV release, (2) fusion with lysosomes for degradation, and (3) fusion with autophagosomes to form amphisomes, which may undergo degradation or, in selected contexts, secretory autophagy. Representative regulators are shown along major routes, including Rab5, Rab11, Rab27a/b, Rab7, ESCRT, and ceramide. Lipid remodeling and tumor-associated stress reprogram trafficking regulation, autophagy flux, and membrane dynamics, thereby reshaping EV release, cargo composition, and heterogeneity. These changes in EV release and cargo composition are linked to downstream consequences relevant to tumor biology, including immune remodeling, metastatic niche conditioning, and therapy resistance. Pathways are simplified and some routes are context-dependent

EV release has often been described as a set of parallel processes involving endosomal secretion, plasma membrane budding, or autophagy-associated export. A more informative view is that EV biogenesis emerges from an interconnected intracellular trafficking network in which vesicle fate is shaped by endosomal sorting, degradative and secretory routing, recycling pathways, and autophagy-associated compartments. In cancer cells, these processes can be further rewired by metabolic, microenvironmental, and therapeutic stress, thereby altering EV composition, release efficiency, and downstream function [6, 16, 17]. This integrated framework is summarized in Fig. 1. Together, endosomal sorting, autophagy coupling, lipid remodeling, and tumor-associated stress help shape EV biogenesis and release.

#### Endosomal fate decisions and route switching under stress

The endosomal trafficking network is initiated at the Rab5-positive early endosome, which serves as a primary sorting hub for internalized cargoes before maturation into multivesicular bodies (MVBs). MVBs occupy a central decision point in EV biogenesis. Some MVBs fuse with lysosomes and enter degradative pathways, whereas others fuse with the plasma membrane and release ILVs as EVs. Recent work further suggests that ILV fate itself can remain dynamic, as a subset of ILVs may undergo retrofusion and re-enter the limiting membrane of MVBs rather than proceeding unidirectionally toward secretion or degradation [18].

This balance is regulated largely by Rab GTPase-dependent trafficking programs. Broadly, Rab7-associated routing favors lysosomal degradation, whereas Rab27a/b supports MVB docking and fusion at the plasma membrane. Recycling endosome-associated pathways, exemplified by Rab11-dependent secretion, may provide alternative secretory routes under selected conditions [6, 19]. These routes should not be interpreted as functionally equivalent in all settings, but they indicate that endosomal trafficking decisions are plastic rather than fixed.

In tumor cells, this plasticity can be accentuated by stress. Metabolic or microenvironmental signals that attenuate Rab7-dependent lysosomal routing have been reported to shift MVBs toward secretion rather than degradation [20–22]. Under nutrient stress such as glutamine deprivation, tumor cells may preferentially engage Rab11a-associated recycling endosome pathways and release EV populations enriched in specific cargoes, including amphiregulin (AREG), with consequences for adaptive signaling and therapy response [23–25]. Live-cell imaging studies further indicate that MVB secretion can be spatially polarized during cell migration, suggesting that vesicle release is not only pathway-dependent but also dynamically organized in space and time [26]. Current evidence indicates that endosomal fate decisions function as adaptive regulators of EV output. However, many of the molecular switches described so far have been defined in selected tumor models, and their generalizability across EV subclasses and solid tumor contexts remains to be established.

#### Autophagy–endosomal crosstalk and selective secretory programs

EV secretion can also intersect with autophagy-associated trafficking programs. In this context, secretory autophagy refers to situations in which autophagy-related compartments do not proceed exclusively toward lysosomal degradation, but instead contribute to extracellular export. A key interface is the amphisome, formed through fusion between autophagosomes and MVBs, which can serve as a platform for cargo secretion [16, 17].

In selected tumor-associated systems, several observations support this model. CD147 has been reported to stabilize GCN2, impair autophagic maturation, and promote amphisome formation, thereby facilitating cargo export through EV-related pathways [27]. Prostate cancer models likewise suggest that CAV1 can be secreted through autophagy-associated vesicular routes, illustrating how non-canonical secretory programs may externalize oncogenic or stromal-remodeling factors [28].

Autophagy-related pathways may also shape selective cargo loading. Regulation of the STX17 axis has been linked to the release of LC3-positive EVs [29], while LC3 itself can participate in LC3-dependent EV loading and secretion (LDELS), functioning outside its canonical degradative role and cooperating with ESCRT machinery and Rab27a to package defined cargoes into vesicles [30]. These findings indicate that autophagy-associated compartments are not exclusively degradative endpoints and may, in some contexts, function as selective secretory platforms.

More specialized structures have recently been proposed. One example is the RAB22A-driven “Rafeesome”, suggested to mediate direct export of endoplasmic reticulum-derived cargoes [31]. The contribution of such emerging pathways to bulk EV production and their relevance across tumor types remain incompletely resolved.

#### Lipid metabolism as a regulatory layer of EV formation and cargo sorting

Lipid metabolism is an additional regulatory layer in EV biogenesis and cargo sorting. Beyond serving as membrane material, lipids can shape membrane curvature, compartment identity, and molecular partitioning. In practical terms, lipid-mediated control of EV formation operates at at least three levels: promotion of membrane deformation and ILV generation, provision of lipid signals that recruit or restrict sorting factors at endosomal membranes, and metabolic support of vesicle production through altered lipid flux [12, 13, 32].

At the level of membrane dynamics, ceramide remains one of the best-characterized lipid regulators of ESCRT-independent ILV formation [33, 34]. Phosphoinositides also help define endosomal membrane identity and sorting behavior. PI3P has been implicated in recruiting selected proteins to MVB-associated membranes [35], whereas stress-responsive changes in PI4P abundance have been reported to recruit Rab10 and promote ILV generation and EV release [36]. These observations suggest that specific lipid species do not simply accompany vesicle formation, but can directly influence the trafficking logic of EV biogenesis.

Lipid-dependent control also extends to cargo selection. Lipid modification of proteins, such as palmitoylation of Claudin7, can influence their entry into secretory endosomal compartments, supporting a role for lipid-associated signals in cargo routing [37]. CD63 has been proposed to direct cholesterol toward ILVs [38], whereas CAV1 can regulate cholesterol availability in selected systems and thereby influence cargo loading, including extracellular matrix-associated proteins such as Tenascin-C [39]. At a broader metabolic level, recent work suggests that fatty acid oxidation-derived NADPH may support cholesterol biosynthesis and thereby provide a permissive metabolic basis for vesicle production [40]. These links between lipid metabolism and EV output are increasingly supported, but the causal contribution of specific lipid species or lipid-metabolic programs to EV subtype formation remains incompletely resolved. In many cases, the relevant mechanisms have been defined in restricted cellular systems rather than broadly across solid tumors.

### Plasma membrane-derived and emerging EV release pathways

**Fig. 2:**
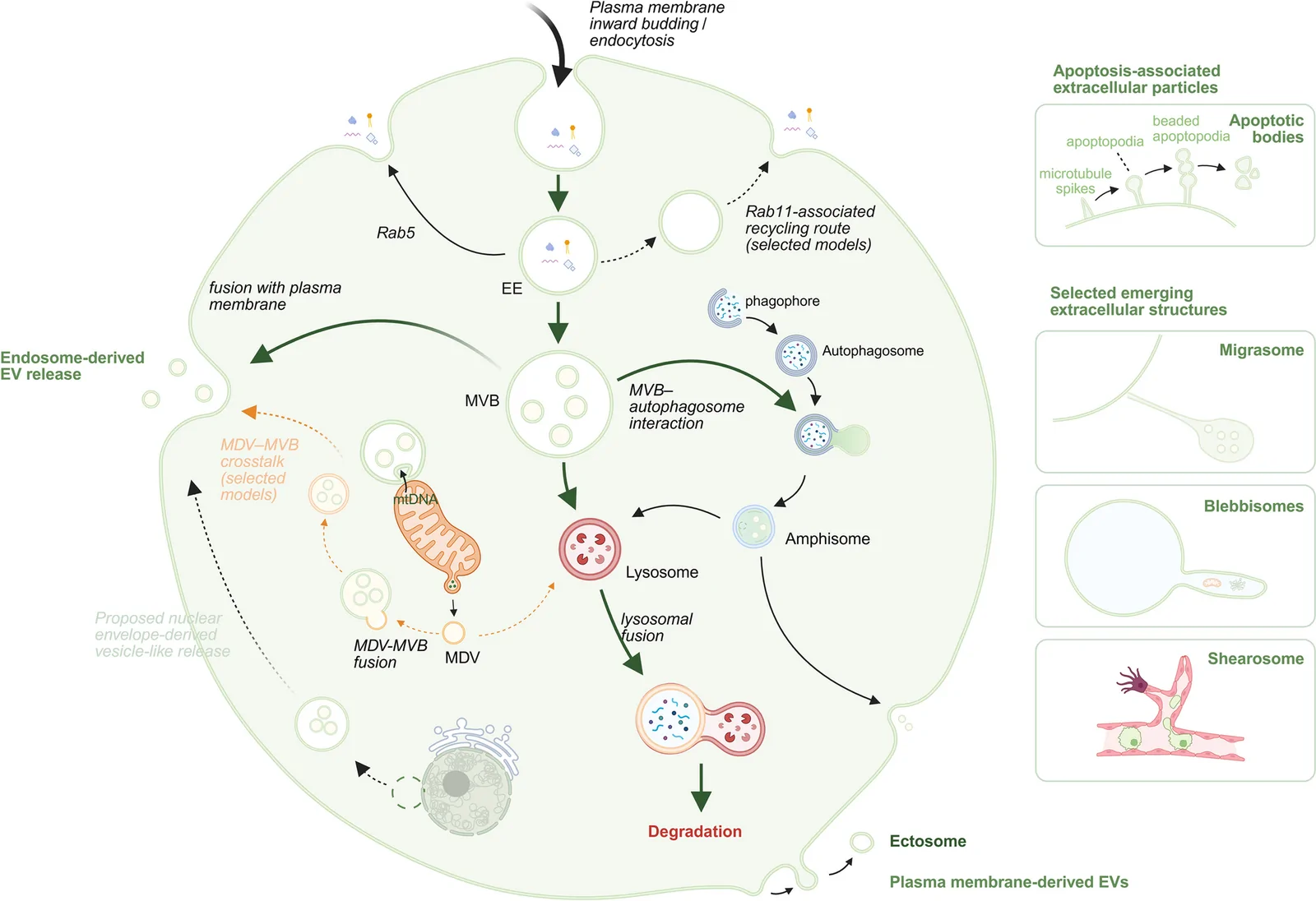
Canonical EV biogenesis pathways and selected related extracellular structures in tumor cells. Schematic overview of canonical EV biogenesis together with selected related extracellular structures reported in tumor-associated settings. (1) Canonical endosome-derived EVs: Plasma membrane inward budding generates early endosomes (EE), which mature into multivesicular bodies (MVBs). Fusion of MVBs with the plasma membrane releases endosome-derived EVs, commonly referred to as exosomes. Selected intracellular route variations shown here include Rab11-associated recycling endosome trafficking, MVB–autophagosome interaction with amphisome formation, lysosomal degradation, and MDV–MVB crosstalk linked to mtDNA-containing EV release in selected models. (2) Plasma membrane-derived and apoptosis-associated particles: Direct outward budding gives rise to ectosomes (microvesicles), whereas apoptosis-associated extracellular particles include apoptotic bodies. (3) Selected related extracellular structures: Additional extracellular structures reported in tumor-related contexts include migrasomes, blebbisomes, shearosomes, and proposed nuclear envelope-derived vesicle-like release. These structures indicate that extracellular release in tumor-associated settings is more diverse than canonical endosome-derived EV pathways alone. Abbreviations: EE, early endosome; MVB, multivesicular body; MDV, mitochondria-derived vesicle; mtDNA, mitochondrial DNA; CTC, circulating tumor cell. Created with BioRender.com

In contrast to endosome-derived small EVs, plasma membrane-derived EVs are generated through direct outward budding or shedding from the cell surface, often in response to specific physiological or pathological cues. These include conventional microvesicles (or ectosomes) [41], as well as vesicles released during programmed cell death, such as apoptotic exosomes (ApoExos) [42–45]. Additional extracellular structures have also been proposed, further expanding the diversity of EV-related release pathways. An overview of canonical EV biogenesis routes and selected related extracellular structures is provided in Fig. 2.

Migrasomes arise during cell migration and, in selected tumor-associated settings, have been implicated in intercellular communication and microenvironmental remodeling, including macrophage polarization in osteosarcoma models [46, 47]. Large extracellular structures such as blebbisomes have been described as carriers of bulky cargoes, including organelles and immune-regulatory proteins such as CD47 [48, 49]. Shearosomes, shed from circulating tumor cells under high shear stress, have likewise been proposed as locally acting mediators that may contribute to pro-metastatic niche remodeling [50, 51]. These observations indicate that extracellular release in cancer is not restricted to canonical endosomal secretion and may include multiple membrane-derived and stress-associated routes.

At the same time, these emerging pathways should not yet be interpreted as equally established counterparts of endosome-derived EV biogenesis. Much of the current evidence still comes from selected cellular systems or specific tumor models, and their definitions, experimental boundaries, and relative contribution to total extracellular vesicle output remain less clearly resolved. Newer extracellular structures such as migrasomes, blebbisomes, and shearosomes are therefore best viewed as conceptually important additions to the landscape of extracellular release, rather than as fully standardized EV categories comparable to canonical endosome-derived pathways.

## EVs in tumor ecosystem remodeling

### EVs in multicellular tumor remodeling

The tumor microenvironment is a dynamic ecosystem composed of malignant cells, stromal populations, vascular networks, and immune infiltrates. Within this setting, EVs act alongside soluble factors, metabolites, and direct cell–cell interactions to shape multicellular communication, particularly in stromal reprogramming and vascular adaptation.

#### Intercellular crosstalk and stromal reprogramming

Within the tumor cell compartment, EVs can mediate horizontal transfer of selected nucleic acids, oncogenic fragments, and signaling complexes, thereby influencing proliferative and survival pathways in recipient cells [52–63]. In selected stem-like cell models, including pancreatic cancer, EVs derived from stem-like populations have been reported to transfer morphogen-associated cargoes to non-stem tumor cells, suggesting a role in maintaining intratumoral heterogeneity in some contexts [64].

EVs also mediate reciprocal signaling between tumor cells and the surrounding stroma, particularly cancer-associated fibroblasts (CAFs). Tumor-derived EVs (TEVs) can promote conversion of resident fibroblasts toward tumor-supportive states through transfer of selected proteins and regulatory RNAs [65–69]. In turn, activated CAFs release EVs enriched in metabolites, non-coding RNAs, proteases, and matrix-remodeling factors that influence tumor-cell metabolism, extracellular matrix organization, and signaling plasticity. In selected models, CAF-EVs have also been linked to epigenetic reinforcement of tumor-promoting feedback loops, including pathways related to TGF-β signaling and extracellular matrix remodeling [70, 71].

These stromal EV circuits may become more consequential under therapeutic stress. CAF-derived EVs have been associated with adaptive resistance through mechanisms involving suppression of mitochondrial apoptosis, modulation of lipid peroxidation, and reduced sensitivity to ferroptotic stress in selected experimental settings [72–75]. Other studies suggest that vesicle-associated proteins such as Annexin A6 may stabilize integrin signaling and activate downstream mechano-transduction pathways, including FAK–YAP, thereby supporting tumor-cell survival during treatment [76]. EV-mediated stromal crosstalk is therefore better viewed as a reciprocal and context-dependent network that can reinforce tumor growth, matrix remodeling, and adaptive fitness.

#### Angiogenesis and vascular permeability

In parallel with stromal remodeling, EVs have been implicated in vascular adaptation that supports tumor growth and dissemination. TEVs can promote angiogenesis by delivering pro-angiogenic cargoes or by reprogramming bone marrow-derived progenitor populations, often under conditions influenced by hypoxia within the primary tumor [77–83]. EV-mediated signaling has also been associated with increased vascular permeability through disruption of endothelial junctional integrity and modulation of pericyte function, thereby facilitating tumor-cell intravasation and extravasation [84–87].

Beyond the blood vasculature, EVs can influence lymphatic remodeling. By transferring regulatory RNAs and proteins to lymphatic endothelial cells, EVs have been reported to activate transcriptional programs such as PROX1 signaling, promoting lymphangiogenesis and lymph node dissemination in selected models [88–92]. These findings support a role for EVs in vascular and lymphatic plasticity within tumors, although the relative contribution of EV-dependent versus non-vesicular signaling is likely to vary across tumor types and microenvironmental contexts.

### EVs in immune regulation

EV-mediated immune regulation is a major component of tumor ecosystem remodeling. In many solid tumors, TEVs contribute to immune suppression by directly impairing effector lymphocytes and by promoting suppressive myeloid and regulatory networks. At the same time, EV-mediated immune effects are not uniformly suppressive. In selected settings—particularly those involving immune-cell-derived vesicles or therapy-induced stress responses—EVs may also support antigen presentation and immune activation. These divergent functions likely contribute to differences in tumor immune phenotype and therapeutic responsiveness.

#### Effector suppression and checkpoint resistance

TEVs can directly impair the function of cytotoxic lymphocytes, particularly CD8⁺ T cells and natural killer (NK) cells, through multiple, non-mutually exclusive mechanisms. One major layer of suppression involves extension of checkpoint signaling beyond direct cell–cell contact. In addition to membrane-bound PD-L1, TEVs may carry other PD-1-axis components, including PD-1 or PD-L2 [93, 94], as well as regulatory factors such as circCCAR1 that are associated with T-cell exhaustion [95]. In hepatocellular carcinoma, tumor-associated macrophages (TAMs) have also been identified as an additional source of PD-L1-positive EVs [96]. Representative mechanisms regulating EV-PD-L1 expression, sorting, and secretion are summarized in Table 1.

**Table 1:** Representative molecular mechanisms regulating EV-PD-L1 expression, sorting, and secretion in tumors

Regulatory Layer	**Key Molecule(s)**	**Mechanistic Effect**	**Cancer Type**	**Ref**
Upstream Signaling	ceRNA: HMGB1/RICTOR	Activates mTORC2-AKT-mTORC1 axis to upregulate PD-L1 transcription	HCC	[97]
Protein Stability	circ-CDYL/HRNR	Stabilizes HRNR protein, leading to mTORC1 activation and PD-L1 upregulation	HCC	[98]
Post-translational Modification	MFGE8/UBE4A	Induces K63-linked ubiquitination of PD-L1, promoting its sorting into EVs	Melanoma, Breast, Lung	[99]
ESCRT-Dependent Sorting	HRS (via ERK phosphorylation)	Phosphorylation of ESCRT component HRS enhances selective PD-L1 loading	Melanoma	[100]
ESCRT-Independent Sorting	GOLM1/Rab27b	Diverts PD-L1 from the plasma membrane to the exosomal pathway	HCC	[101]
Protein–Protein Interaction	STAM2 (ESCRT-0 component)	Acts as a bridge, binding PD-L1 and recruiting HRS for EV loading	OSCC	[102]
Phase Separation	DDX10/Rab27b	Forms condensates via liquid–liquid phase separation (LLPS) to promote PD-L1 EV secretion	OSCC	[103]
Cytokine Signaling	IFN-γ	Induces high-level production of exosomal PD-L1 as an adaptive immune resistance mechanism	Melanoma	[104]

*Abbreviations*: *EVs* extracellular vesicles, *PD-L1* programmed cell death-ligand 1, *HCC* hepatocellular carcinoma, *OSCC* oral squamous cell carcinoma, *ESCRT* endosomal sorting complex required for transport, *ceRNA* competitive endogenous RNA, *mTOR* mammalian target of rapamycin, *LLPS* liquid–liquid phase separation, *IFN-γ* interferon-gamma

TEV-mediated suppression also extends beyond canonical checkpoint pathways. TEV-associated CD73 has been linked to local adenosine generation [105, 106], while other studies suggest that TEVs may perturb intracellular signaling or early T-cell activation through selected cargos, including miR-424-mediated inhibition of the CD28–CD80/86 co-stimulatory axis [107–112].

A further layer of dysfunction involves metabolic paralysis of effector lymphocytes. In multiple metastatic models, lipid-rich EV populations released within the tumor ecosystem have been reported to induce lipid overload in recipient NK cells and CD8⁺ T cells, resulting in profound functional exhaustion [113, 114]. Similarly, PD-L1-bearing TEVs have been associated with cholesterol synthesis, lipid droplet accumulation, and senescence-like dysfunction in T cells [115]. Additional TEV cargoes can deepen this phenotype. For example, circUHRF1 has been shown to upregulate TIM-3 on NK cells [116], and TEVs have also been linked to indirect NK-cell dysfunction through macrophage-dependent exhaustion pathways involving the PVR–TIGIT axis [117].

**Fig. 3:**
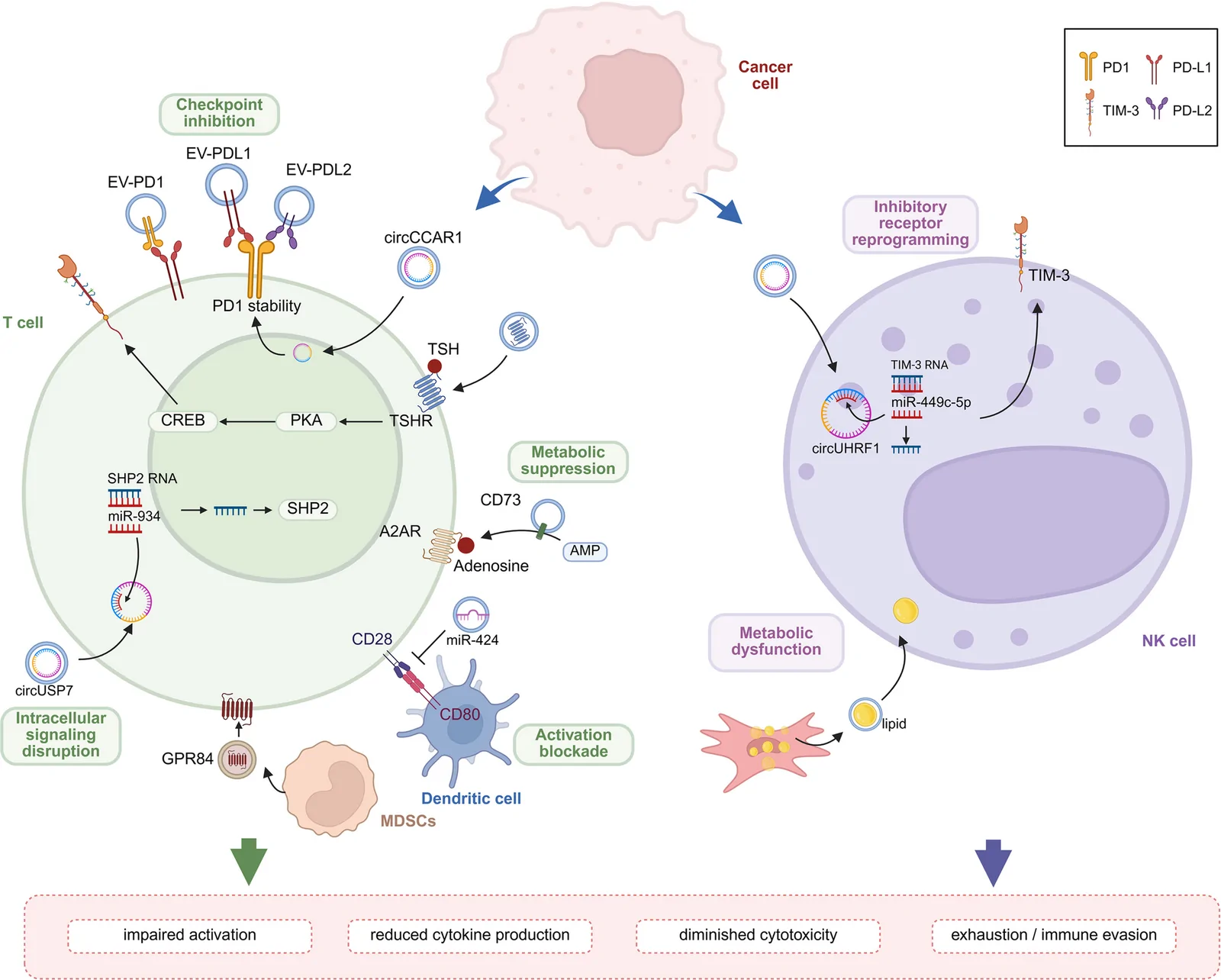
Representative EV-mediated mechanisms of effector immune-cell suppression. This figure summarizes representative mechanisms by which tumor-derived and selected host-derived extracellular vesicles (EVs) suppress effector immune-cell function, with CD8⁺ T cells and natural killer (NK) cells as the principal targets. On the T-cell side, EV-mediated suppression includes extension of PD-1-axis signaling, adenosine-associated metabolic suppression, inhibition of CD28–CD80/86 co-stimulation, and interference with intrinsic signaling programs. On the NK-cell side, representative mechanisms include TIM-3-associated inhibitory reprogramming, lipid-associated metabolic dysfunction, and impaired cytotoxicity. Together, these EV-mediated effects contribute to impaired activation, reduced cytokine production, diminished killing capacity, and enhanced exhaustion/immune evasion. Mechanisms shown are representative rather than exhaustive, and their relative contribution is likely to vary across tumor types and experimental settings

These convergent mechanisms have direct implications for resistance to immune checkpoint inhibitors (ICIs). EV-associated PD-L1 can function as a circulating decoy that reduces the effective activity of anti-PD-1/PD-L1 therapies, while other TEV cargos may continue to suppress T-cell function even when receptor blockade is pharmacologically engaged [112]. This resistance is not purely tumor-cell-autonomous. Host- and stromal-derived EVs can also reinforce T-cell dysfunction and therapeutic refractoriness, including in obesity-associated settings and through tumor-associated macrophage-derived EV programs that limit CD8⁺ T-cell infiltration or impair antigen presentation [107, 118–120]. Overall, the role of EVs in effector-cell dysfunction and ICI resistance is increasingly supported, but many of the specific mechanisms described so far remain defined in selected experimental systems and may not operate uniformly across all solid tumors. Representative mechanisms of EV-mediated effector immune-cell suppression are summarized in Fig. 3.

#### Suppressive niche formation and regulatory recruitment

**Fig. 4:**
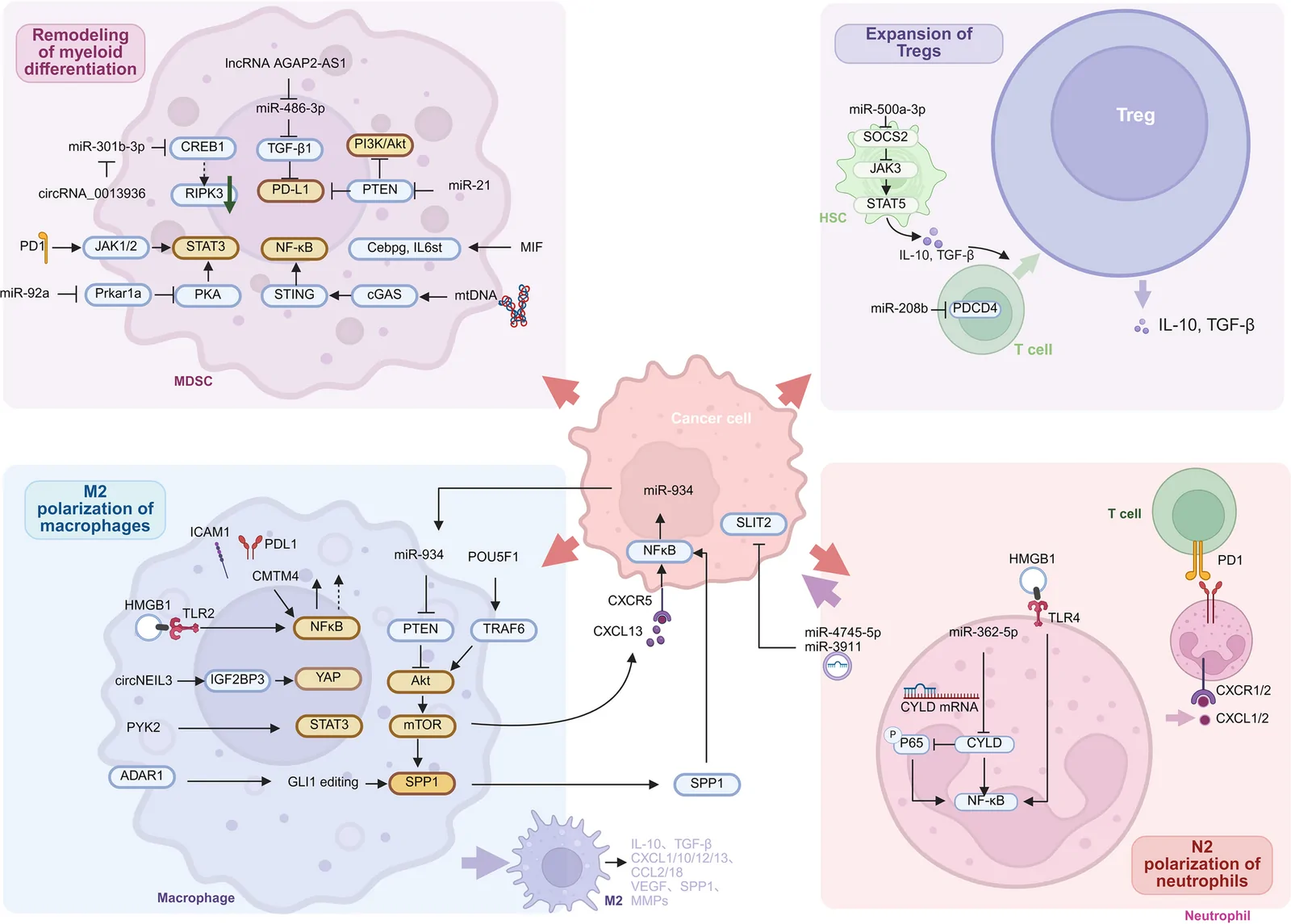
Induction of an immunosuppressive cellular network by tumor-derived extracellular vesicles (TEVs). TEVs contribute to the establishment of an immunosuppressive microenvironment by reprogramming multiple immune-cell populations. (1) M2 polarization of macrophages: EV cargoes activate representative downstream signaling and metabolic programs, including pathways related to PI3K/AKT, STAT3, NF-κB, and mTOR, thereby promoting M2-like polarization and reinforcing suppressive feedback loops in selected models. (2) N2 polarization of neutrophils: EV-associated DAMPs and regulatory RNAs can activate inflammatory signaling, including NF-κB-related pathways, and promote the emergence of immunosuppressive N2-like tumor-associated neutrophil states. (3) Remodeling of myeloid differentiation: EVs can skew myeloid-cell development by promoting MDSC expansion while impairing differentiation toward functionally competent dendritic cells. (4) Expansion of regulatory T cells (Tregs): EVs may support Treg expansion either directly or indirectly by establishing IL-10/TGF-β-rich tolerogenic niches through stromal and immune reprogramming. Together, these effects help organize a broader suppressive cellular network within the tumor ecosystem. Mechanisms shown are representative rather than exhaustive, and their relative contribution is likely to vary across tumor types and experimental settings. Abbreviations: TEVs, tumor-derived extracellular vesicles; TAMs, tumor-associated macrophages; TANs, tumor-associated neutrophils; MDSCs, myeloid-derived suppressor cells; DCs, dendritic cells; Tregs, regulatory T cells. Created with BioRender.com

Beyond directly suppressing effector lymphocytes, TEVs also contribute to the establishment of suppressive immune niches by reshaping myeloid differentiation, regulatory lymphocyte expansion, and local metabolic conditions. These effects may occur both systemically and within the tumor microenvironment, where TEVs have been implicated in altering hematopoietic progenitor differentiation and in skewing the lineage polarization of multiple immune subsets [121] (Fig. 4).

Within the innate immune compartment, TEVs promote suppressive myeloid reprogramming, including polarization of macrophages toward an M2-like state [122–124] (Table 2), induction of N2-like neutrophil phenotypes [130–132], and expansion of myeloid-derived suppressor cells (MDSCs) [133]. Metabolic rewiring appears to reinforce this process. Under selected stress conditions, tumor cells have been reported to release EVs enriched in lipid species that are preferentially taken up by macrophages and MDSCs, leading to intracellular lipid accumulation and reinforcement of suppressive phenotypes. In some models, this process has been associated with foam-cell-like transitions in tumor-associated macrophages [134–138]. TEVs may also reprogram endogenous metabolic circuitry in recipient myeloid cells, including pathways linked to fatty acid oxidation, fatty acid transport, and cholesterol biosynthesis, while EV-associated lipids may function not only as cargoes but also as extracellular metabolic substrates [128, 133, 139–141]. These findings indicate that EVs contribute to immunosuppressive metabolic flux within the tumor ecosystem.

**Table 2:** Representative mechanisms of macrophage reprogramming by tumor-derived EVs

**Cargo Class/Key Cargo**	Cancer Type	**Core Mechanism of Reprogramming**	**Pro-tumoral Consequence**	**Ref**
Translational Machinery	Colorectal Cancer (CRC)	Delivers functional protein synthesis machinery to boost translation of M2-associated genes	Promotes an immunosuppressive microenvironment	[125]
ADAR1 Protein	Colorectal Cancer (CRC)	Edits AZIN1 RNA to promote polyamine synthesis, driving M2 polarization	Induces oxaliplatin resistance via SPP1 secretion	[126]
HMGB1 (DAMP molecule)	NSCLC	Drives metabolic reprogramming via the TLR2/NF-κB axis to promote PD-L1 synthesis	Creates an immunosuppressive pre-metastatic niche	[127]
CD36 Protein	Ovarian Cancer	Induces a senescent, lipid-laden macrophage (SnLLM) phenotype by enhancing lipid uptake	Remodels the omental microenvironment to promote metastasis	[128]
miR-9-5p	Breast Cancer	Remotely reprograms hepatocyte cholesterol metabolism to polarize Kupffer cells toward an M2 phenotype	Establishes a hepatic pre-metastatic niche	[129]

References are cited according to the main bibliography. "Translational machinery" refers to the horizontal transfer of proteins and/or RNAs involved in protein synthesis. SnLLM represents a specialized subpopulation of macrophages induced in the TME that supports tumor progression

*Abbreviations*: *EVs* extracellular vesicles, *CRC* colorectal cancer, *NSCLC* non-small cell lung cancer, *M2* M2-polarized (pro-tumoral) macrophages, *ADAR1* adenosine deaminase acting on RNA 1, *SPP1* secreted phosphoprotein 1 (also known as osteopontin), *HMGB1* high mobility group box 1, *DAMP* damage-associated molecular pattern, *TLR2* Toll-like receptor 2, *NF-κB* nuclear factor kappa-light-chain-enhancer of activated B cells, *PD-L1* programmed cell death-ligand 1, *SnLLM* senescent, lipid-laden macrophage

At the adaptive-immune level, TEVs have also been linked to expansion of regulatory T cells (Tregs), either directly through lineage differentiation or indirectly by conditioning stromal and myeloid compartments to generate cytokine-rich tolerogenic niches [142, 143]. TEVs may simultaneously limit the initiation of adaptive immunity by interfering with myeloid progenitor development and suppressing the differentiation and function of dendritic cells (DCs) [144, 145]. Suppressive niche formation therefore appears to involve convergent effects on myeloid skewing, metabolic reinforcement, Treg expansion, and impaired antigen priming rather than a single dominant EV pathway. Although the overall role of EVs in this process is increasingly supported, many of the specific metabolic and lineage-reprogramming mechanisms remain based on selected experimental systems and may not be uniformly conserved across solid tumors.

#### Immune-cell-derived EV broadcasting

Immune cells within the tumor microenvironment are not merely passive recipients of tumor-derived EV signals, but can themselves act as active EV broadcasters capable of transmitting both suppressive and anti-tumor signals. Tumor-associated macrophages (TAMs), in particular, can disseminate vesicular signals that act directly on tumor cells or indirectly through stromal intermediates. For example, TAM-derived EVs have been reported to transfer DOCK7 to recipient tumor cells, promoting cholesterol efflux, increasing plasma membrane fluidity, and enhancing migratory behavior and liver metastasis in selected models [146]. TAM-derived EVs can also participate in higher-order relay circuits: EV-associated NAMPT released by TAMs has been shown to reprogram nicotinamide metabolism in cancer-associated fibroblasts (CAFs), which in turn influences functional exhaustion of infiltrating CD8⁺ T cells [147]. These findings indicate that immune-cell-derived EVs can propagate tumor-supportive signals both through direct tumor–immune interactions and through multicellular relay mechanisms involving stromal intermediates.

T cells likewise contribute to EV-based immune broadcasting. Regulatory T cells (Tregs), in addition to secreting soluble suppressive mediators, release EVs enriched in the ectonucleotidases CD39 and CD73. These vesicles can convert extracellular ATP into immunosuppressive adenosine and thereby extend suppressive effects beyond the immediate vicinity of the parent Treg cell, weakening nearby dendritic cells and effector T cells. This form of EV-associated adenosinergic suppression is not restricted to Tregs. Under chronic antigenic stimulation, effector CD8⁺ T cells have also been reported to release CD73-rich EVs, generating a cell-free negative-feedback loop that dampens local immune responses [148].

Importantly, immune-cell-derived EVs are not uniformly pro-tumorigenic. Certain effector T-cell subsets can also broadcast anti-tumor signals through EVs. For example, CD45RO⁻ CD8⁺ T cells have been shown to secrete EVs enriched in miR-765 that directly target tumor cells. In endometrial cancer models, transfer of these vesicles suppresses estrogen-driven tumor progression through modulation of the Notch signaling axis [149]. Resident immune-cell-derived EVs therefore represent a heterogeneous and functionally bidirectional layer of communication within the tumor microenvironment.

#### Immune activation under selected conditions

Although most studies have focused on immunosuppressive EV programs, EV-mediated immune effects are not exclusively suppressive. Under selected conditions, EVs can support anti-tumor immunity through at least three non-exclusive routes: amplification of antigen presentation by professional antigen-presenting cells, immune activation mediated by lymphocyte-derived EVs, and therapy-induced conversion of TEVs toward immunogenic signaling outputs.

At the level of antigen presentation, EVs released by professional antigen-presenting cells can amplify host immune responses. High-resolution intravital imaging has shown that, within tumor-draining lymph nodes, migratory dendritic cells (DCs) carrying tumor antigens can form specialized immune synapses with resident DCs and transfer antigen-loaded vesicles across the local DC network. This vesicle-mediated antigen sharing expands the number of antigen-presenting nodes and can enhance CD8⁺ T-cell cross-priming [150]. These findings suggest that APC-derived EVs may function not merely as passive carriers of tumor material, but as intermediates that extend antigen presentation across local APC networks. Lymphocyte-derived EVs can also contribute to immune activation. Activated CD4⁺ T cells have been reported to release EVs containing functional interferon-γ (IFNγ), which, upon uptake by macrophages, sensitize myeloid cells to STING activation, promote inflammatory polarization, and help overcome innate immune tolerance [151]. This indicates that EV-mediated communication can, in some contexts, reinforce rather than suppress myeloid immune activation.

The immunological properties of TEVs can also be reshaped by environmental and therapeutic context. Following radiation or chemotherapy-induced DNA damage, stressed tumor cells may release EVs enriched in cytosolic double-stranded DNA (dsDNA). Once taken up by dendritic cells, these DNA-containing vesicles can act as danger-associated signals that activate the cGAS–STING pathway, drive type I interferon production, and support subsequent T-cell cross-priming [152]. Emerging evidence further suggests that such immunogenic EV signaling may extend beyond the local tumor bed. Irradiated tumors have been reported to release microparticles enriched in mitochondrial DNA (mtDNA), which can be taken up by splenic neutrophils and activate a STING/NLRP3 inflammasome axis, thereby enhancing systemic dendritic-cell antigen presentation and distal cytotoxic T-cell responses [153]. In addition, therapy-stressed tumor cells may actively disseminate immune-stimulatory signals through rewired vesicular trafficking pathways. One example involves RAB22A-associated non-canonical autophagy, in which activated STING complexes are packaged into specialized compartments and secreted in EV populations reported to propagate interferon-driven anti-tumor signaling within the tumor microenvironment [154].

These findings indicate that EV-mediated immune effects are more context-dependent than a strictly suppressive model would imply. However, many of the immunostimulatory programs described so far have been defined in specific treatment settings or experimental systems, and their generalizability across solid tumors remains to be established.

### EVs in metastatic niche formation and cross-organ adaptation

**Fig. 5:**
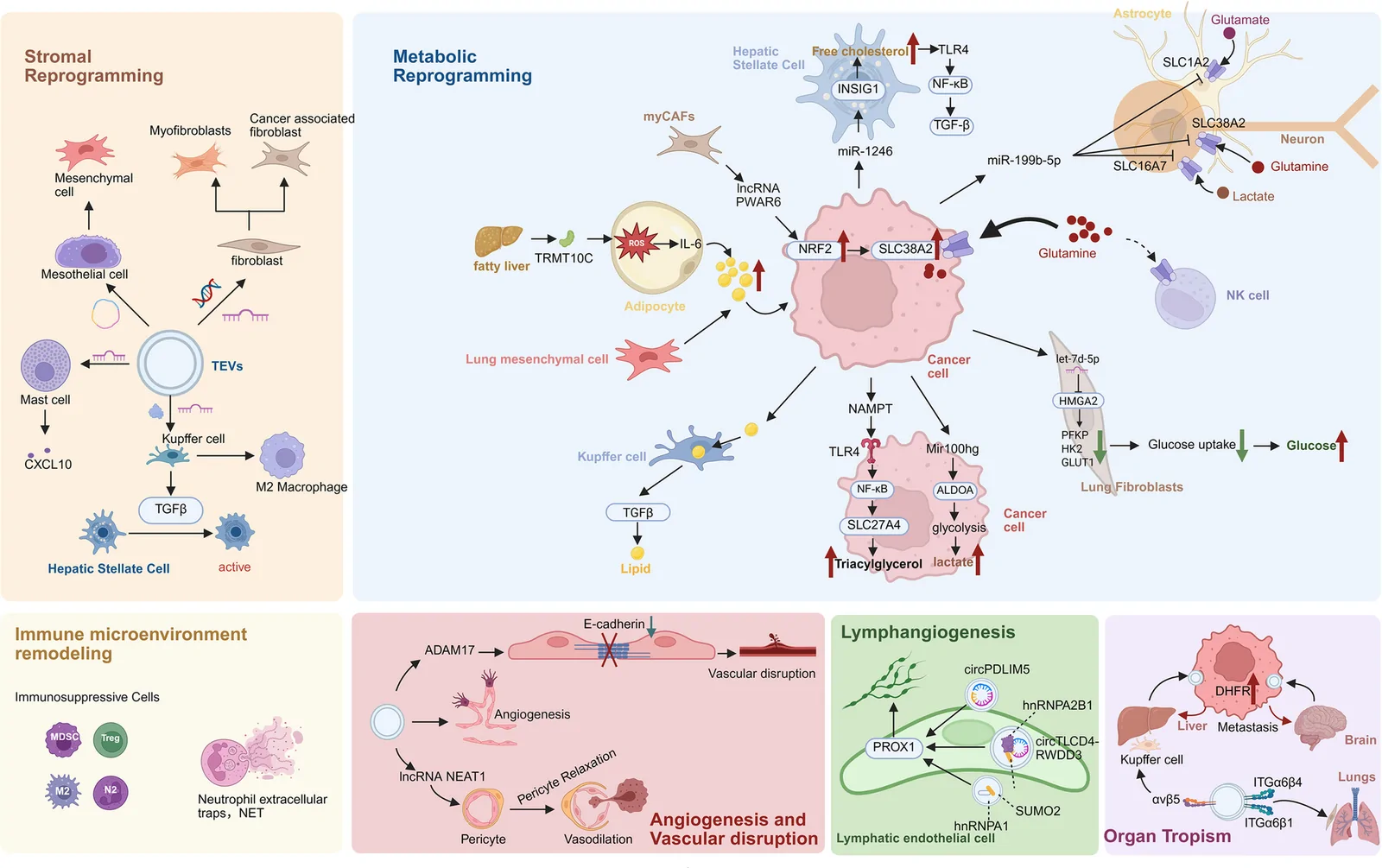
EV-mediated remodeling during the cancer metastatic cascade. Schematic overview of representative EV-mediated processes during metastatic progression. Tumor-derived EVs (TEVs) contribute to metastatic dissemination by remodeling both the primary tumor microenvironment and distant organ niches. At local and systemic levels, EV-mediated signaling promotes stromal reprogramming, metabolic rewiring, immune microenvironment remodeling, angiogenesis and vascular disruption, lymphangiogenesis, and organotropic niche conditioning, thereby creating conditions favorable for tumor-cell dissemination and survival. In distant tissues, EVs interact with stromal, vascular, immune, and tissue-resident cells to help establish pre-metastatic niches (PMNs). In selected contexts, organ-derived EVs may also provide feedback signals that further enhance the metastatic competence of tumor-cell subpopulations, contributing to a self-reinforcing communication loop between the primary tumor and target organs. The convergence of EV-primed tumor cells and permissive distant niches ultimately facilitates metastatic seeding, colonization, and outgrowth. Mechanisms shown are representative rather than exhaustive, and the relative contribution of individual EV-mediated processes is likely to vary across tumor types, metastatic sites, and experimental settings. Abbreviations: EVs, extracellular vesicles; TEVs, tumor-derived extracellular vesicles; PMNs, pre-metastatic niches; CTCs, circulating tumor cells; NETs, neutrophil extracellular traps. Created with BioRender.com

Recent advances suggest that metastasis is not merely a passive consequence of tumor-cell dissemination, but a process of systemic tissue remodeling in which EVs mediate communication between primary tumors and distant organs. However, much of the mechanistic evidence remains organ- and model-dependent (Fig. 5).

#### Organotropism and pre-metastatic niche initiation

Metastatic dissemination begins with organotropism, in which the integrin repertoire on EV membranes helps direct vesicle trafficking to specific organs—for example, α6β4/α6β1 to the lung and αvβ5 to the liver [155–157]. Once localized, TEVs can initiate early adhesion–penetration programs that promote circulating tumor cell (CTC) retention and disrupt local tissue barriers [158–160]. This process is not purely unidirectional. Once primed, target organs may release their own EVs as recruitment cues that favor attraction and survival of compatible CTC populations [161–163]. This reciprocity is exemplified by metabolically altered organs such as the fatty liver, which can secrete EVs that reshape tumor signaling and promote local immunosuppressive adaptation, thereby favoring colonization in selected models [164, 165]. Pre-metastatic niche initiation therefore appears to involve reciprocal host–tumor communication rather than a purely one-way process of invasion.

#### Stromal, immune, and vascular remodeling of the pre-metastatic niche

Following organ-specific homing, TEVs can further participate in multidimensional remodeling of distant tissues, promoting their transition toward a pre-metastatic niche (PMN) more permissive for metastatic outgrowth. One major dimension of this process involves reprogramming of resident stromal cells. In liver metastasis models, TEV-associated macrophage migration inhibitory factor (MIF) has been reported to induce TGF-β secretion from Kupffer cells, which in turn activates hepatic stellate cells and promotes local fibronectin deposition [166]. Similar multicellular cascades have been described in brain metastasis settings, where EV-exposed astrocytes release miRNA-rich vesicles that facilitate recruitment of immunosuppressive microglia, thereby contributing to a permissive pre-metastatic ecosystem [167].

TEVs can also contribute to immune and inflammatory conditioning of the PMN. Multiple models suggest that TEVs transfer lipids and other pro-inflammatory signals to tissue-resident immune cells, including Kupffer cells, inducing cytokines such as TNF-α and IL-6 and promoting a chronic low-grade inflammatory environment that may favor subsequent immunosuppressive niche formation [168–170]. TEVs have also been implicated in induction of neutrophil extracellular traps (NETs), which in selected settings may help circulating tumor cells evade cytotoxic T-cell clearance [171, 172]. In addition, macrophages reprogrammed by TEV signaling have been associated with platelet aggregation and microthrombus formation, potentially providing physical support for CTC arrest and metastatic seeding [173].

Vascular and metabolic preconditioning provide additional layers of PMN remodeling. TEVs have also been linked to increased angiogenesis and vascular permeability at distant sites, although the contribution of these effects appears to vary across organs and models [78–92]. At the metabolic level, TEV-associated post-transcriptional regulators such as miR-9-5p have been reported to disrupt cholesterol and oxysterol homeostasis in Kupffer cells, a change associated with pro-tumor macrophage polarization and establishment of a relatively immune-permissive metastatic niche [129]. PMN formation appears to involve coordinated stromal, immune, vascular, and metabolic reprogramming rather than a single dominant pathway. At the same time, the relative contribution of these mechanisms is likely to vary substantially across metastatic organs and tumor contexts.

#### Cross-organ feedback and systemic coordination

Emerging evidence suggests that metastatic progression involves reciprocal feedback between primary tumors and target organs. Once exposed to TEVs, primed distant tissues may themselves release EV populations into the circulation, where they function as recruitment or selection cues for compatible CTC subpopulations. In this sense, metastatic dissemination is not simply a one-way process of tumor-cell spread, but part of a broader cross-organ communication loop in which host tissues actively participate. This feedback network may be amplified by tumor-intrinsic states. PTEN-deficient tumors, for example, have been reported to hypersecrete EVs, increasing the burden of circulating pro-metastatic signals and intensifying systemic niche priming [174]. The architecture and strength of these feedback loops are likely to vary across tumor types and host contexts, and many remain defined in selected experimental systems.

### EVs in stress adaptation and therapy resistance

Tumor progression requires continuous adaptation to environmental and therapeutic stress, and EVs may participate in this process by linking stress-associated trafficking changes to metabolic communication and resistance phenotypes.

#### Metabolic rewiring under endogenous stress

Even before therapy, tumors must adapt to hypoxia, nutrient limitation, acidosis, and oxidative imbalance. As discussed in Sect. "EV Biology: Structure, Biogenesis, and Functional Diversity", such stresses can alter EV biogenesis and cargo selection. The focus here is not on stress responses per se, but on how stress-adapted EV programs may reshape metabolic interactions within the tumor ecosystem. Under stress, EV output may shift toward secretory programs that favor export of adaptive cargoes. In selected settings, nutrient, hypoxic, and oxidative stress have been associated with selective loading of stress-relevant or immunomodulatory molecules into EVs [117, 175, 176]. Metabolic byproducts such as lactate, together with altered endosomal trafficking, may redirect multivesicular bodies away from lysosomal degradation and toward secretion, thereby coupling intracellular stress to broader microenvironmental signaling outputs [21, 177]. These findings are most consistent with a model in which EV release becomes part of a stress-adaptive secretory program rather than a constitutive background process.

Stress-adapted EV programs may also facilitate local and, in some contexts, systemic metabolic redistribution. Altered lipid synthesis and cholesterol flux are often reflected in the EV lipidome, suggesting that EVs can act as carriers of metabolic resources under nutrient-limited conditions. Through transfer of energy-rich lipids or selected metabolic enzymes, EVs may support nutrient-stressed tumor cells while also contributing to metabolic competition or dysfunction in neighboring immune populations [82, 113, 178–180]. In some settings, TEVs may further contribute to systemic metabolic redistribution, including adipose tissue lipolysis and disruption of hepatic glucose homeostasis, while selected stromal EV populations have also been implicated in sustaining dormant or nutrient-stressed tumor cells through metabolically supportive cargoes [181–183]. Viewed together, these studies place EVs within broader local and systemic metabolic adaptation networks under stress, although the extent to which such programs operate across tumor types and disease settings remains uncertain.

#### EV-associated resistance networks under therapeutic pressure

Under therapeutic pressure, EV-mediated communication may contribute to adaptive resistance states within the tumor microenvironment. Rather than functioning as uniformly dominant drivers, EVs are better interpreted as one component of a broader multicellular defense network that can facilitate resistance through signal transfer, microenvironmental remodeling, and metabolic buffering. At the molecular level, EVs have been implicated in horizontal transfer of resistance-associated signals. In selected models, EV cargoes can activate compensatory survival pathways in recipient cells and thereby support dissemination of therapy-tolerant phenotypes [184–187]. Drug stress has also been associated with release of mitochondrial-derived vesicles (MDVs) under certain treatment conditions, while other studies suggest that TEVs may act as molecular decoys by binding therapeutic nanoparticles or engaging CAR-T cells through surface-associated molecules [188–191]. These findings indicate that EVs can participate in direct propagation of resistance-associated signals, although the relative contribution of any single mechanism is likely to vary substantially across treatment contexts.

Therapeutic stress may also reinforce resistance indirectly by reshaping the surrounding microenvironment. Drug exposure has been reported to induce apoptotic tumor cells or stromal cells to release EVs enriched in pro-survival factors such as Annexin A6 [76, 192, 193]. In selected settings, these vesicles have been linked to increased matrix deposition by cancer-associated fibroblasts, which may restrict drug penetration [194], or to shifts in immune-cell polarization toward more suppressive states [141, 195, 196]. Such findings argue against a purely tumor-cell-autonomous view of resistance and instead support a broader interpretation in which therapy-induced EV release helps stabilize protective niches for residual disease.

A further layer of adaptation involves metabolic buffering against treatment-induced cell death. In models of chemotherapy-associated lipid peroxidation, including ferroptosis-related stress, EVs derived from adipocytes or tumor cells have been reported to transfer lipid transporters or metabolic enzymes that reduce the intracellular abundance of vulnerable lipid species [197, 198]. Similarly, EV-mediated remodeling of cholesterol metabolism, including stabilization of the cholesterol esterification enzyme ACAT1, has been implicated in transfer of cisplatin-resistant traits within the microenvironment [199]. These mechanisms are biologically plausible and increasingly supported, but they remain best viewed as emerging, context-dependent layers of resistance rather than uniformly established programs across cancers.

### Non-tumor EV sources in cancer ecology

Tumor ecology is not limited to communication among tumor cells and host-derived stromal or immune populations. Increasing evidence suggests that microbiome-derived extracellular vesicles, including bacterial extracellular vesicles (bEVs) and, in Gram-negative bacteria, outer membrane vesicles (OMVs), add a cross-species layer to EV-mediated tumor communication. Their significance lies less in any single microbial product than in the broader possibility that microbial EVs may influence systemic immune tone, local immune states, and responsiveness to immunotherapy. At present, however, this field remains more emerging and model-dependent than the corresponding literature on tumor- or host-derived EVs.

One major pattern involves *systemic conditioning through the gut–tumor axis*. In selected models, bEVs released by commensal bacteria have been reported to cross the intestinal barrier, reach peripheral immune organs, and modulate anti-tumor immunity. For example, OMVs derived from *Akkermansia muciniphila* have been associated with enhanced responses to PD-1 blockade, while bEVs from *Bacteroides fragilis* or *Bifidobacterium* species have been linked to altered NK-cell activity or PD-1 sensitivity in selected settings [200–202]. A second pattern involves local niche modulation by tumor-resident or translocated bacteria. In this context, bEVs have been implicated in induction of PD-L1, suppression of macrophage antimicrobial functions, or maintenance of relatively “cold” immune states, as reported for *Porphyromonas gingivalis*, *Klebsiella pneumoniae*, and *Fusobacterium nucleatum* in selected tumor-associated models [203–205].

At the same time, microbiome-derived EVs should not be viewed as uniformly tumor-promoting. Some bacterial EV populations appear capable of enhancing inflammatory or anti-tumor immune responses, and in selected preclinical settings have shown adjuvant-like activity, including promotion of antigen-specific CD8⁺ T-cell responses or activation of γδ T cells [206–210]. The emerging picture is therefore not one of a simple pro-tumor versus anti-tumor dichotomy, but of strong functional polarity shaped by microbial composition, cargo identity, recipient-cell context, and host state. For this reason, microbiome-derived EVs are best regarded at present as an important but still exploratory dimension of tumor ecology—one that expands current models of tumor ecology, while also requiring considerable caution in interpretation and generalization.

### Context-dependent functional polarity of EVs

EV function is not fixed, but varies with cargo composition, cellular origin, and recipient context. This plasticity complicates biological interpretation and helps explain why EV effects often resist broad generalization across models. This context dependence is illustrated by the divergent effects of related cargoes and processes. Tumor-derived EV-associated mitochondrial DNA (mtDNA) can promote immunosuppressive programming in MDSCs [211, 212], whereas genomic DNA (gDNA) delivered to Kupffer cells activates antitumor innate immunity [213]. Donor-cell identity adds a further layer of polarity: although TEVs are more often associated with tumor-promoting functions, MEK1-containing EVs have also been reported to activate macrophages and induce antitumor effects in selected settings [214]. Similarly, a process that appears protective in the donor cell may acquire opposite consequences after transfer; extrusion of tumor-suppressive miRNAs, for example, can become immunosuppressive when these molecules are repurposed by recipient cells [215]. The microenvironment can also reshape EV output, as TEVs may support immune surveillance under normoxia but shift toward immune evasion under hypoxia [216]. EVs thus operate along a context-dependent functional continuum rather than within a strictly pro-tumoral or anti-tumoral framework.

## EV biomarkers in cancer liquid biopsy

Extracellular vesicles (EVs) are widely present in biofluids, including blood and urine, and can stably carry nucleic acids, proteins, and lipids derived from their cells of origin. These features have made EVs attractive candidates for cancer liquid biopsy. Compared with conventional single-analyte biomarkers, EVs may provide multidimensional molecular information and, to some extent, capture dynamic changes in tumor biology. EVs have accordingly been investigated for early detection, prognostic assessment, treatment monitoring, and resistance prediction. However, the clinical utility of EV-based biomarkers will depend on whether they can be reproducibly measured, interpretable across complex biological samples, and consistently validated across analytical platforms.

### The evolving diagnostic paradigm

The development of EV-based diagnostics has paralleled a broader shift in oncology from single-marker detection toward multiparametric and systems-oriented interrogation. Early assays often focused on individual analytes, including GPC1 and early EV-associated clinical assays such as ExoDx® Lung (ALK) [217]. However, the marked spatial and temporal heterogeneity of solid tumors soon highlighted the limitations of relying on single EV-associated markers. This has driven the field toward multiparameter models (Table 3), including clinically advanced platforms such as the FDA-designated ExoDx® Prostate test, which integrates a multigene signature, and composite approaches such as MedOncAlyzer™, which combine EV analytics with ctDNA [218–223]. EVs have shown potential for early detection, disease monitoring, progression assessment, and resistance prediction in solid tumors, but clinical implementation remains constrained by insufficient standardization in EV isolation, characterization, and downstream analysis.

**Table 3:** Representative EV-based diagnostic products with clinical or regulatory development

Product Name (Trade Name)	Disease/Indication	Clinical Application/Purpose	Biomarkers/Technical Principle	Sample Type	Company	Regulatory/Clinical Deployment Status
ExoDx™ Lung(ALK)	Non-Small Cell Lung Cancer (NSCLC)	Guide ALK inhibitor targeted therapy	exoRNA: Detection of EML4-ALK fusion gene	Plasma	Exosome Diagnostics (a Bio-Techne brand)	CLIA-certified Laboratory Developed Test (LDT)
ExoDx® Prostate (IntelliScore, EPI)	Prostate Cancer	Aid in diagnosis; Risk stratification to avoid unnecessary biopsies	exoRNA (3-gene panel): ERG, PCA3, and SPDEF mRNA expression levels integrated into a risk score via an algorithm	Urine	Exosome Diagnostics (a Bio-Techne brand)	FDA Breakthrough Device Designation (2019)
Human Exosomal CA125, HE4, and C5a Detection Kit (Misyou™)	Epithelial Ovarian Cancer	Aid in diagnosis; Risk assessment	Exosomal proteins (3-protein panel): Quantitative detection of CA125, HE4, and C5a to calculate an Ovarian Cancer Score (OCS) (Chemiluminescence Immunoassay)	Serum	3DMed Diagnostics	China NMPA Approved (No. 20243400138)
Human Neural Exosome α-synuclein Diagnostic Kit (Paliming™)	Parkinson's Disease	Aid in diagnosis	Neural exosomal protein: Qualitative detection of α-synuclein (Magnetic Particle Chemiluminescence Immunoassay)	Plasma	KAI XIANG HONG KANG	Beijing MPA Approved (No. 20252400610)

Regulatory status reflects the highest level of approval/clearance as of January 2026. China NMPA approval No. 20243400138 and Beijing MPA approval No. 20252400610 represent significant regulatory milestones for EV-based protein assays in clinical oncology and neurology

*Abbreviations*: *ALK* anaplastic lymphoma kinase, *NSCLC* non-small cell lung cancer, *CLIA* Clinical Laboratory Improvement Amendments, *LDT* laboratory developed test, *ERG* ETS-related gene, *PCA3* prostate cancer antigen 3, *FDA* U.S. Food and Drug Administration, *NMPA* National Medical Products Administration (China), *MPA* Medical Products Administration, *α-synuclein* alpha-synuclein

This evolution also changes what EV liquid biopsy is expected to capture. The central question is no longer simply whether a specific molecule can be detected, but whether EV-associated signals can be used to interpret tumor-related ecosystems more dynamically. This is particularly relevant in immuno-oncology, where single-site tissue biopsy may not adequately capture multicellular signaling states or systemic immune adaptation over time. In this context, circulating EVs may offer a complementary window into dynamic tumor–host interactions. EV diagnostics are therefore more likely to depend on integration of multi-origin signals, longitudinal monitoring, and stricter methodological control than on isolated markers alone.

### EV biomarkers for immune phenotyping and ICI response

In precision immuno-oncology, EV liquid biopsy is increasingly being explored as a means of assessing tumor immune states and predicting responses to immune checkpoint inhibitors (ICIs). Compared with single-site tissue biopsy, circulating EVs may offer a more systemic and dynamic perspective, thereby complementing current approaches to immune profiling. At the same time, the analytical maturity of this field varies substantially across biomarkers, tumor types, and detection platforms. The central challenge, therefore, is to distinguish EV-associated markers that are approaching clinical utility from those that remain biologically informative but analytically immature [224].

Among immune-related EV biomarkers, circulating EV-PD-L1 remains the most extensively studied and arguably the closest to clinical translation. Several studies suggest that baseline levels and early on-treatment changes in EV-PD-L1 may reflect adaptive interactions between tumors and the host immune system and may correlate with response to anti-PD-1/PD-L1 therapy [104]. Even so, interpretation of this biomarker requires caution. Its origin may not be restricted to tumor cells, and differences in detection platform, EV isolation workflow, and quantification strategy can substantially affect comparability [225]. EV-PD-L1 is therefore better regarded as a promising dynamic immune biomarker that still requires multicenter and standardized validation, rather than as a universally established clinical standard.

Beyond EV-PD-L1, the field is increasingly moving toward composite EV signatures that aim to capture broader immune states rather than a single checkpoint axis. For example, EVs derived from cancer-associated fibroblasts and enriched in THBS2 have been associated with T-cell exclusion and relatively “cold” tumor states in selected studies [226]. Likewise, immune-cell-derived EV populations may provide complementary stratification value. Immunocyte-derived PD-1/CD80⁺ small EVs have been linked to cold tumor phenotypes and adaptive immunosuppression, and their combined analysis with circulating PD-L1 signals has been reported to help distinguish responders from non-responders to anti-PD-1 therapy [227]. Similarly, EV-PD-1 [228] and EV-PD-L2 [229] have been proposed as complementary checkpoint-related axes. These directions are biologically attractive because they may better reflect multicellular immune states and patient stratification than single-analyte readouts. However, most remain supported primarily by selected cohorts or tumor models and should not yet be interpreted as broadly generalizable clinical standards.

A similar principle applies to EV-associated metabolic markers. Exosomal CD73 has been proposed as an indicator of adenosine-related immune suppression [105], while combined modeling of exosomal lactate with EV-PD-L1 and related immune markers has been reported to improve prediction of objective response rate (ORR) or progression-free survival (PFS) in selected settings [230]. These findings support the idea that metabolically informed EV signatures may help characterize immunosuppressive microenvironments beyond checkpoint molecules alone. At present, however, such metabolic–immune composite models remain early-stage, and their apparent performance is likely to depend heavily on cohort composition, sample handling, and analytical methodology.

Multimodal integration and artificial intelligence (AI) are also beginning to shape EV-based predictive models, but they are better viewed at present as exploratory frameworks than as mature clinical infrastructure. Existing studies have begun to integrate EV features with radiomics, non-coding RNA profiles, and other multimodal signals to improve prediction of ICI response [231–234]. Nonetheless, machine-learning models in this space remain limited by modest sample sizes, batch effects, limited feature interpretability, and insufficient generalizability across independent cohorts [224]. More realistically, AI is likely to be most useful only when complex, low-abundance EV-derived signals can first be analytically defined and reproducibly measured. Its clinical value will therefore depend less on algorithmic sophistication alone than on pre-analytical standardization, external validation, and cross-platform reproducibility.

### Challenges in defining tumor-specific EV biomarkers

The central difficulty in defining tumor-specific EV biomarkers is not a single technical limitation, but the combined problem of source ambiguity, EV heterogeneity, co-isolated confounders, and insufficiently standardized measurement systems. In circulation, tumor-derived EVs (TEVs) usually represent only a small fraction of the total particle pool and coexist with particles derived from normal tissues, immune cells, platelets, and other non-tumor sources. Recent multi-omics studies of human plasma small EVs indicate that circulating samples contain both relatively conserved EV-associated protein and lipid features and distinguishable non-EV particle features, underscoring that detection of a signal does not by itself establish tumor-EV specificity [235]. Host-related variables such as age and sex can further influence plasma EV and protein-enriched fractions, adding another layer of biological variability to biomarker interpretation [236].

EV heterogeneity introduces a second, conceptually distinct obstacle. Single-particle analyses have shown that canonical tetraspanins such as CD9, CD63, and CD81 are not uniformly distributed across individual EVs, and that capture strategies based on selected tetraspanins can alter the apparent sensitivity of multiplexed biomarker detection and distort the inferred composition of EV subpopulations [237]. In practical terms, the EVs “seen” by a given platform may not fully correspond to the EV populations actually present in the sample. Although cotargeting multiple tetraspanins has been proposed as one way to improve analytical sensitivity, such approaches have thus far been validated mainly in restricted models and platforms and still require broader evaluation in clinical samples [238]. Heterogeneity is therefore not only a biological feature of EV populations, but also an analytical problem that shapes what can be detected and how biomarker signals are interpreted.

This ambiguity is compounded by the uncertain molecular origin of many detected signals. Reference proteomic studies have shown that proteins repeatedly identified in small EV preparations do not necessarily represent bona fide vesicle-encapsulated cargo, but may instead derive from co-isolated non-vesicular components, protein aggregates, or externally associated materials [235, 239]. Among the most persistent confounders in blood-derived EV studies are lipoproteins. Because EVs and lipoproteins overlap in size, density, and some physicochemical properties, co-isolation is common in conventional workflows [2, 240]. Importantly, the problem extends beyond residual contamination alone. Emerging evidence suggests that EVs and lipoproteins may also engage in context-dependent binding or form functional complexes, further altering apparent particle identity and affecting downstream analysis [241, 242]. In plasma and serum studies, lipoproteins should therefore be treated not merely as background noise, but as variables that directly influence EV definition, quantification, and functional interpretation.

A further bottleneck lies in measurement science itself. Lack of harmonized standards for EV separation, characterization, and quantification continues to weaken comparability across studies. MISEV2023 explicitly recommends stricter frameworks for EV terminology, isolation, characterization, and functional analysis [2], while subsequent discussion surrounding MISEV updates makes clear that the field is still converging on what constitutes sufficient evidence for EV identity and rigor [243]. Even widely used platforms such as flow cytometry can yield markedly different EV concentration measurements across instruments, although calibrated workflows have been shown to improve inter-platform comparability and support the establishment of reference ranges [244]. A similar problem applies to EV-RNA biomarkers, where differences in RNA extraction, library preparation, sequencing platform, and bioinformatic pipeline can substantially alter apparent results and limit transferability across cohorts [245]. For this reason, even EV-associated miRNAs, lncRNAs, or isomiRs that appear highly informative within single studies should be interpreted cautiously until supported by broader analytical validation. Integration of MISEV principles with molecular quantification frameworks such as MIQE has accordingly been proposed to strengthen the analytical validity of EV-RNA biomarker research [225].

Taken together, these issues indicate that the main barrier to clinical translation is no longer candidate discovery alone, but the ability to define, measure, and validate EV-derived signals under analytically complex conditions. The critical problem is not simply limited sensitivity, but the difficulty of identifying biomarkers that are simultaneously tumor-informative, biologically interpretable, and technically reproducible in heterogeneous clinical samples. Movement from “proof-of-potential” toward measurable, comparable, and regulatable clinical implementation remains constrained by inadequate measurement science, incomplete quality-control frameworks, and unresolved regulatory classification [246]. The field therefore needs not only additional candidate markers, but also orthogonal characterization workflows, quantification of co-isolated components, clearer analytical boundaries, and rigorous validation across multicenter cohorts.

## Source selection and scalable manufacturing in EV engineering

Clinical translation of engineered EVs depends on two closely linked decisions: selecting an appropriate producer-cell source and establishing scalable, reproducible manufacturing pipelines. The parent cell shapes vesicle identity in ways that influence tropism, immunogenicity, bioactivity, and manufacturability. Source selection, purification, quality control, formulation, and process design therefore jointly shape the therapeutic identity of the final EV product. Because translational progress remains constrained by incomplete mechanistic understanding, insufficient standardization, and unresolved regulatory classification [246, 247], source choice and process design should be viewed as risk-weighted translational decisions rather than purely technical optimizations.

### Selecting the producer-cell source

Selecting an appropriate producer cell is a foundational decision in EV engineering because vesicle phenotype is strongly shaped by the parent cell. In practice, source choice requires balancing at least three competing dimensions: inherent biological function, including therapeutic activity and native targeting behavior; manufacturability, including yield, scalability, and batch-to-batch consistency; and clinical safety, including immunogenicity and oncogenic risk. No currently available source simultaneously maximizes therapeutic function, targeting precision, manufacturability, and biosafety. Source selection should therefore be treated as an application-specific translational trade-off rather than a search for a universally superior platform [246, 248].

Current EV engineering strategies draw on four broad source categories. One uses cells with intrinsic therapeutic activity in an effort to generate cell-free analogues of cell therapy. EVs derived from immune cells, such as NK cells or M1 macrophages, may retain cytotoxic or immunomodulatory properties [249–252], whereas stem-cell-derived EVs may provide regenerative or anti-inflammatory functions [253–255]. However, the same biological complexity that makes these sources attractive can also complicate batch comparability, mechanism-of-action definition, and potency assessment. A second strategy exploits the innate trafficking properties of specific cell types. TEVs may display strong homotypic homing in selected settings [256–260], whereas macrophage- or neutrophil-derived EVs may traffic to inflamed tissues and, in some models, cross the blood–brain barrier [261–267]. Such advantages, however, may be offset by cargo liability, inflammatory activity, or broader product-safety concerns.

A third strategy uses relatively reduced-risk or biologically simplified platforms as engineering chassis. Anucleated red blood cell-derived EVs, for example, offer favorable biosafety features [268–271], whereas immortalized lines such as HEK293T provide scalable and genetically tractable production systems [272]. These platforms may be advantageous for manufacturing, but may also sacrifice some degree of intrinsic biological specialization. A fourth, more exploratory direction investigates non-mammalian sources, including plant-derived vesicles and bacterial EVs, to exploit potential advantages such as scalability, oral delivery, or intrinsic adjuvanticity. At present, however, these systems remain less mature than mammalian platforms and raise unresolved questions regarding compositional standardization, product definition, and regulatory evaluation [246]. For MSC-related secretome products in particular, recent translational analyses emphasize that cell sourcing, preconditioning, expansion strategy, comparability, dose definition, and validated potency assays must be considered as an integrated development problem rather than as separable optimization steps [248]. A detailed comparison of these sourcing strategies is provided in Table 4, with plant-derived examples summarized separately in Table 5.

**Table 4:** Comparative overview of cellular sources for engineered EV production

Strategy	Source	Core Properties	Key Advantages	Key Challenges	Manufacturability	Safety Profile	Best-Fit Application	Ref
Sources with Intrinsic Therapeutic Activity	Mesenchymal Stem Cells (MSCs)	Inflammation-homing; Regenerative & immunosuppressive	Potential for allogeneic or standardized production; Innate therapeutic effects	Functional dichotomy; Batch inconsistency	High (scalable 3D culture)	Good (low immunogenicity)	Drug delivery; Regenerative therapy; Mitigating toxicity	[253, 273, 274]
	Dendritic Cells (DCs)	Rich in pMHC-I/II & co-stimulatory molecules	Potent T-cell priming; Natural lymph node homing	Low yield; Requires ex vivo antigen loading/maturation	Low (autologous)	Excellent (autologous)	Personalized Cancer Vaccines	[275–277]
	Natural Killer (NK) Cells	Carries cytotoxic cargo (Perforin, Granzymes); Killer ligands	Direct tumor killing; Broad cytotoxicity; Low GvHD risk	Moderate yield; Finite lifespan (for primary cells)	Medium (from NK cell lines, e.g., NK-92)	Good	"Off-the-shelf" Cytotoxic Therapy	[249–251, 278–280]
	M1-Polarized Macrophages (M1-Φ)	Pro-inflammatory payload (e.g., miRNAs) that reprograms TME	TME modulation; Reverses immunosuppression	Polarization state stability; Source availability	Low-Medium	Good (autologous)	TME Reprogramming (e.g., M2-to-M1 switch)	[252]
	Engineered T/NK Cells (CAR-EVs)	Surface CAR for precision targeting; Carries cytotoxic payload	CAR precision with superior safety profile (e.g., mitigated CRS/GvHD risk)	Production scalability; Ensuring CAR loading on EVs	Medium–High (from cell lines)	Good–Excellent (non-replicating)	"Off-the-shelf" Targeted Immunotherapy	[281–284]
Sources with Intrinsic Targeting Properties	Tumor Cells	Reported homotypic and heterotypic tumor tropism	Potent tumor targeting; Personalized vaccine source	High oncogenic risk; Innately immunosuppressive	Low-Medium	Poor (tumorigenic risk)	Target discovery; High-risk delivery (requires engineering)	[256–260, 285]
	Macrophages/Neutrophils	TME tropism; BBB-crossing ability	Natural delivery to brain tumors & inflamed niches	Source availability; State-dependent function	Low-Medium	Good (autologous)	CNS drug delivery; Targeting inflammatory metastases	[261–267]
Reduced-Biological-Complexity Engineering Chassis	Red Blood Cells (RBCs)	Anucleated; Biologically inert	Favorable biosafety profile; Abundant & standardized source	No intrinsic functions; 100% reliant on engineering	Very High (From blood banks)	Excellent (Minimal immunogenicity)	Versatile drug/RNA delivery platform	[268–271]
	Engineered Lines *e.g., HEK293T*	High growth & transfection rates	widely used research and production platform; High yield; Genetically tractable	Non-therapeutic origin; Potential immunogenicity/contaminants	Very High (industrial scale)	Moderate (requires stringent QC)	R&D platform; Large-scale EV production	[272]
Non-Mammalian Source Platforms	Plants *e.g., Grape, Ginger*	Intrinsic bioactivity; Cross-kingdom regulation	Extreme safety (no zoonosis); Agricultural-scale cost & production	Oral bioavailability; Unforeseen immune response	Very High	Excellent (presumed)	Oral delivery; Nutraceuticals; Microbiome modulation	[286–296]
	Milk	Oral stability (GI-resistant)	Abundant & sustainable; Proven oral bioavailability	Profound functional dichotomy (e.g., can inhibit primary tumor but accelerate metastasis via TGF-β2)	Very High	Good	Oral drug/siRNA delivery	[297–305]
	Bacteria (BEVs)	Potent innate immune stimulation (PAMPs)	Powerful intrinsic adjuvant; Scalable & engineerable	Endotoxin risk (LPS); Systemic inflammation risk	Very High (fermentation)	Poor (native state)	Vaccine-oriented immunotherapy applications; Immune agonists; "Cold-to-hot" TME conversion	[202, 209, 306–312]

Manufacturability and safety assessments are qualitative, literature-informed comparisons rather than standardized rankings. Functional dichotomy in milk- and MSC-derived EVs refers to their context-dependent pro- or anti-tumoral effects. References are cited as they appear in the main bibliography

*Abbreviations*: *EVs* extracellular vesicles, *MSCs* mesenchymal stem cells, *DCs* dendritic cells, *pMHC-I/II* peptide-major histocompatibility complex class I/II, *NK* natural killer, *GvHD* graft-versus-host disease, *M1-Φ* M1-polarized macrophages, *TME* tumor microenvironment, *miRNAs* microRNAs, *CAR-EVs* chimeric antigen receptor-engineered extracellular vesicles, *CRS* cytokine release syndrome, *BBB* blood–brain barrier, *CNS* central nervous system, *RBCs* red blood cells, *HEK293T* human embryonic kidney 293 T cells, *BEVs* bacterial extracellular vesicles, *PAMPs* pathogen-associated molecular patterns, *LPS* lipopolysaccharide, *QC* quality control

**Table 5:** Summary of plant-derived EVs (PDEVs) for therapeutic applications

Source Plant	Extraction Method	Vesicle Properties & Designation	Target Cells	Loaded Cargo/Drug	Loading Method	Citation
Cabex/Rabex	Ultrafiltration combined with size-exclusion chromatography (SEC)	Exosome-like nanovesicles (Cabex/Rabex)	Colon cancer cells	Doxorubicin	Incubation, followed by ultrafiltration to remove free drug	[313]
Asparagus (*A. cochinchinensis*)	Differential centrifugation followed by sucrose density gradient ultracentrifugation	nanovesicles	Hepatocellular carcinoma cells	Not applicable (utilized intrinsic bioactivity)	Not applicable	[291]
*Ginger (Zingiber officinale)*	Differential centrifugation followed by sucrose density gradient ultracentrifugation	Exosome-like nanovesicles	Specific gut microbiota; indirect target: melanoma cells (via metabolite DHA)	Endogenous microRNA: aly-miR159a-3p	Utilized endogenous cargo. Reloading of isolated RNA was performed via ultrasonication for validation	[292]
Lemon (*Citrus limon*)	Differential centrifugation followed by sucrose density gradient ultracentrifugation	nanovesicles	Human lung carcinoma (A549) and colorectal adenocarcinoma (SW480) cells	Not applicable (utilized intrinsic antitumor activity)	Not applicable	[289]
Bitter Melon (*M. charantia*)	Electrophoresis- and dialysis-based methods	EVs	Oral squamous cell carcinoma (OSCC) cells	Not applicable (utilized to reduce 5-FU resistance)	Not applicable	[290]
Grapefruit (Citrus paradisi)	Differential centrifugation	Biomimetic EV-DNs: A complex of GEVs and doxorubicin-loaded nanoparticles	Glioma cells	Doxorubicin	Surface patching/conjugation of drug-loaded nanoparticles onto the GEV surface	[314]
Grapefruit (Citrus paradisi)	Differential centrifugation	Transferrin-modified EVs	Multiple tumor cells (4T1, A549, HepG2)	Tri-functional platinum(IV) conjugate	Wrapping method (encapsulation of drug-loaded liposomes into GEVs via ultrasonication)	[315]
Ginseng (*Panax ginseng*)	Differential centrifugation followed by sucrose density gradient ultracentrifugation	Exosome-like nanoparticles (GENs); contain endogenous ginsenosides and other bioactive compounds	Breast cancer cells (4T1)	Endogenous bioactive compounds (e.g., ginsenosides)	Not applicable	[316]
Tea flowers (*Camellia sinensis*)	Differential centrifugation coupled with sucrose density gradient ultracentrifugation	exosome-like nanovesicles	Metastatic breast cancer cells (MCF-7, 4T1)	Endogenous bioactive contents, such as EGCG, ECG, EC, gallic acid, myricetin, etc	Endogenous bioactive contents, such as EGCG, ECG, EC, gallic acid, myricetin, etc	[317]

"Not applicable" in the Loaded Cargo/Drug column indicates that the study utilized the intrinsic biological activity or endogenous therapeutic molecules of the plant vesicles without exogenous drug loading. Vesicle Designation (e.g., Cabex, Rabex, GENs) follows the specific nomenclature established by the cited authors. Surface patching refers to the exogenous attachment of nanoparticles or ligands to the vesicle membrane, while Wrapping typically involves the encapsulation of larger complexes such as liposomes within the EV lumen. References are listed as they appear in the main bibliography

*Abbreviations*: *PDEVs*: plant-derived extracellular vesicles, *SEC* size-exclusion chromatography, *EVs* extracellular vesicles, *DHA* docosahexaenoic acid, *5-FU* 5-fluorouracil, *GEVs* grapefruit-derived extracellular vesicles, *GENs* ginseng-derived exosome-like nanoparticles, *EGCG* epigallocatechin gallate, *ECG* epicatechin gallate, *EC* epicatechin

### Isolation, purification, characterization, storage, and scale-up

**Fig. 6:**
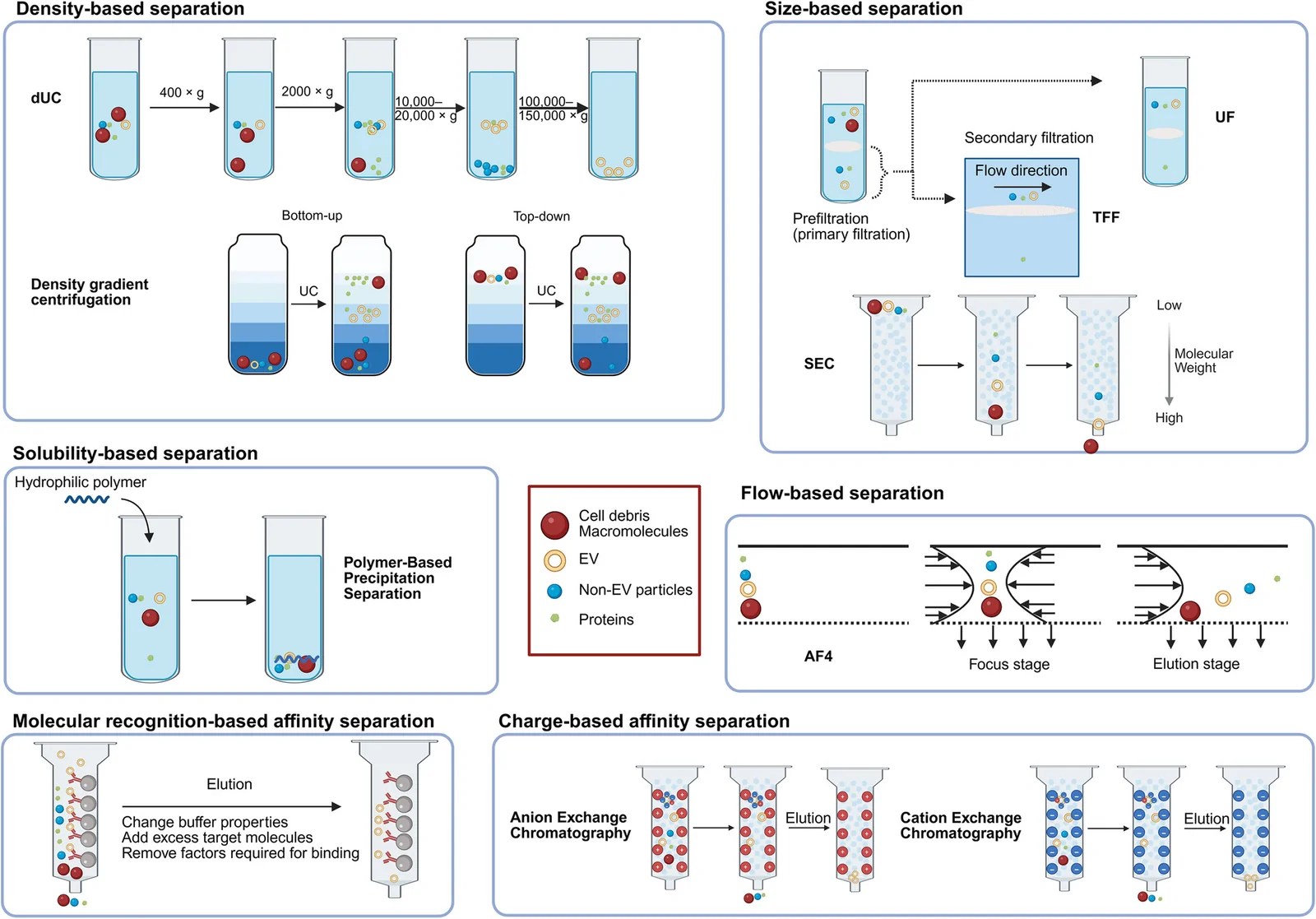
Technical strategies and separation principles for EV isolation. Schematic summary of current technical strategies for EV isolation and purification, categorized by separation principle. (1) Density-based methods: Separation via centrifugal force, including differential ultracentrifugation (dUC) for pelleting and density gradient centrifugation (DGC) for enrichment within specific density ranges (e.g., sucrose, iodixanol). (2) Size-based methods: Fractionation based on hydrodynamic radius/volume, including ultrafiltration, tangential flow filtration (TFF) for continuous concentration, and size-exclusion chromatography (SEC). (3) Solubility-based methods: Hydrophilic polymer-based precipitation (e.g., PEG) to reduce EV solubility. (4) Affinity-based methods: High-specificity capture utilizing either surface charge (anion/cation exchange chromatography) or molecular recognition (probes such as antibodies, aptamers, peptides, or heparin). Target EVs are captured on matrices (e.g., magnetic beads) and subsequently eluted by altering buffer conditions or adding competitive ligands. (5) Flow/field-based methods: Label-free separation using external fields, such as asymmetric flow field-flow fractionation (AF4), which separates EV subpopulations in a coupled cross-flow and laminar flow field. Abbreviations: EVs: extracellular vesicles; dUC: differential ultracentrifugation; TFF: tangential flow filtration; SEC: size-exclusion chromatography; AF4: asymmetric flow field-flow fractionation. Created with BioRender.com

Clinical translation of EV therapeutics requires manufacturing workflows that are not only scalable, but also sufficiently reproducible to support product definition, comparability, and release testing. Because no single isolation method can simultaneously optimize yield, purity, bioactivity, and scalability across all EV sources and intended applications, the field is increasingly moving away from single-method solutions and toward hybrid, product-specific workflows. In practice, this often involves pairing a scalable concentration step, such as tangential flow filtration (TFF), with a higher-resolution purification step, such as size-exclusion chromatography (SEC) or affinity-based separation [318, 319], as summarized in Fig. 6. This shift is consistent with broader efforts to frame EV production around orthogonal and measurement-aware workflows rather than universal processing pipelines [246, 247, 320].

Even within this emerging framework, two bottlenecks remain especially important. The first is purification. Effective removal of co-isolated contaminants with overlapping physical properties, particularly lipoproteins, remains a major barrier to product purity [240, 241, 321–323]. The second is characterization. Bulk measurements alone are often insufficient to resolve the marked heterogeneity of EV preparations, meaning that product identity and batch comparability cannot be established through aggregate measurements alone. Single-vesicle and orthogonal characterization approaches are therefore increasingly regarded as important complements for product definition and comparability, although they are not yet fully standardized as standalone quality-control tools [2, 320, 324, 325]. More broadly, critical quality attributes, potency assays, and release criteria for EV products remain incompletely standardized, which continues to limit regulatory readiness [246, 326]. Functional assays that jointly assess EV integrity and bioactivity are also emerging as useful complements to physicochemical characterization for evaluating batch reproducibility and storage-related change, but such approaches remain product- and platform-dependent [327].

Efforts to improve EV manufacturing now span the entire workflow, from upstream cell expansion to downstream formulation and storage. Upstream strategies aim to increase yield and reduce exogenous contamination through 3D bioreactor systems and serum-free, xeno-free culture conditions [328–332], while technologies such as cellular nanoporation are being explored to support scalable production of cargo-engineered producer cells [333]. Downstream, formulation strategies such as lyophilization are being investigated to improve storage stability [334]. None of these elements should yet be regarded as universally transferable across EV products, because process modifications that improve scalability or stability may also alter composition, functionality, or comparability. The translational challenge is therefore not simply to assemble an end-to-end pipeline, but to establish one in which purification, characterization, formulation, and storage are jointly validated for the specific EV product under development [246, 247, 335]. A systematic comparison of key technologies and their limitations is summarized in Table 6.

**Table 6:** Technical comparison of EV CMC-relevant processing and characterization approaches

Technological Aspect	Technology Category	Specific Method/Strategy	Purity	Yield	Scalability	Cost/Complexity	Key Advantages	Key Limitations & Challenges	Ref
Separation & Purification	Density-Based	Differential Ultracentrifugation (dUC)	Low	Medium	Low	Medium	"Gold standard"; mature tech	Low throughput; Co-isolates contaminants; Risk of vesicle damage	[336–338]
		Density Gradient Centrifugation (DGC)	High	Low	Low	High	Can separate particles of similar density	Time-consuming; Harsh conditions; Low recovery	[339, 340]
	Size-Based	Ultrafiltration/Dead-End Filtration (UF)	V. Low	Varies	High	Low	Simple, fast; easily scalable	Prone to membrane clogging; risk of vesicle damage and loss	[337, 341]
		Tangential Flow Filtration (TFF)	Medium	High	V. High	High	Highly scalable for industry; gentle	Requires specialized equipment & optimization	[342–344]
		Size Exclusion Chromatography (SEC)	Medium	Medium	Medium	Low-Medium	Gentle (preserves bioactivity); Removes small proteins	Dilutes sample; Poor separation from HDL	[339, 345–347]
	Solubility-Based	Polymer-Based Precipitation (e.g., PEG)	V. Low	High	High	V. Low	Simple, fast; high throughput	Unsuitable for functional/clinical use; Polymer carryover	[348, 349]
	Flow/Field-Based	Free Flow Electrophoresis (FFE)	High	Low	Low	V. High	High resolution; gentle and label-free	Low throughput; expensive and complex equipment; charge-dependent	[350, 351]
		Asymmetric Flow Field-Flow Fractionation (AF4)/Field-Flow Fractionation (FFF)	V. High	Low	Low	V. High	High separation resolution; gentle, preserves EV integrity	Complex instrumentation; low throughput; requires parameter optimization	[352–354]
	Affinity-Based	Charge-Based Affinity (e.g., Ion Exchange Chromatography, IEC)	V. High	Low	Low	V. High	High selectivity; can differentiate EV subpopulations; gentle and scalable; fast	Adsorption losses; depends on charge differences; harsh elution conditions	[355–357]
		Molecular Recognition-Based Affinity	V. High	Low	Low	V. High	High specificity; can directly capture from complex biofluids without extensive pre-processing	Single-ligand capture may miss subpopulations; harsh elution conditions; high cost of high-purity ligands; non-specific binding; risk of bead carryover	[358–362]
	Microfluidic Integrated Platforms	Microfluidics	High	V. Low	V. Low	High	Automated; minimal sample volume; excellent efficiency	Low preparative throughput; high chip cost and manufacturing complexity; risk of channel clogging	[363–368]
Quality Characterization	Bulk Analysis	NTA, Western Blot (WB)	/	/	/	Low	Rapid overall assessment	Cannot resolve heterogeneity	[2, 369]
Storage & Transport	Single-Vesicle Analysis	Nanoflow Cytometry (nFCM), TEM	/	/	/	High	Resolves heterogeneity	Technically demanding; Throughput limitations	[369–371]
	Storage Conditions	−80 °C Cryopreservation	/	/	/	Low	Standard method for long-term storage	Requires cold chain; Damage from freeze–thaw	[372, 373]
		4 °C Short-Term Storage	/	/	/	/	Convenient, less damaging than freezing	Short-term storage; bioactivity declines over time; requires low-adhesion tubes to prevent loss	[373–375]
	Stability Enhancement	Addition of Cryoprotectants	/	/	/	/	Improves stability during cryopreservation; reduces aggregation and adsorption loss	Requires cold chain; protection is limited; may introduce exogenous substances	[376–379]
		Lyophilization (Freeze-Drying)	/	/	/	Medium	Ambient temperature storage; can be formulated as an inhalable powder	Risk of aggregation and damage; Requires formulation dev	[380–384]
		Spray Drying	/	/	/	High	Rapid & scalable process; Suitable for inhaled powders	Risk of thermal & shear stress damage; Low recovery; High equipment cost	[385]

Purity, Yield, Scalability, and Cost/Complexity are qualitative comparative ratings based on current bioprocessing standards: V. Low: Very Low; V. High: Very High. "/" indicates that the parameter is not applicable or is highly dependent on the specific downstream application rather than the technology itself. HDL contamination remains a major challenge for size-based methods due to overlapping size distributions with small EVs. References are cited as they appear in the main bibliography

*Abbreviations*: *dUC* differential ultracentrifugation, *DGC* density gradient centrifugation, *UF* ultrafiltration, *TFF* tangential flow filtration, *SEC* size-exclusion chromatography, *HDL* high-density lipoprotein, *PEG* polyethylene glycol, *FFE* free-flow electrophoresis, *AF4* asymmetric flow field-flow fractionation, *IEC* ion-exchange chromatography, *NTA* nanoparticle tracking analysis, *nFCM* nano-flow cytometry, *TEM* transmission electron microscopy, *QC* quality control

### EV heterogeneity, product definition, and classification

The fundamental heterogeneity of EVs continues to complicate their translation into reproducible biopharmaceutical products. This diversity challenges a core assumption of pharmaceutical development: that a product can be clearly defined and consistently manufactured. Two aspects of heterogeneity are especially problematic. First, vesicles originating from different biogenetic pathways often overlap extensively in size, density, and related physical properties, meaning that no single separation method can yield a truly pure subpopulation. Second, the biochemical marker landscape remains ambiguous; canonical markers such as tetraspanins (e.g., CD63 and CD9) are neither exclusive to a single subtype nor uniformly expressed, contributing to a long-standing classification dilemma [386–388]. Recent reference studies have further shown that some commonly detected components of small EV preparations may derive from non-vesicular fractions rather than bona fide vesicle cargo, further complicating product definition [235, 239]. Recent analyses have framed EV heterogeneity as a double-edged property: it creates functional and diagnostic opportunity, yet also impedes standardization, product definition, and therapeutic translation [389]. These challenges have prompted efforts to develop more refined operational and functional classification strategies, including combinatorial marker logic and candidate subtype-enriched markers such as Annexin A1 [387, 390], although no single framework has yet resolved the classification problem comprehensively.

In response to these difficulties, the field is moving away from rigid biogenesis-based categorization toward more pragmatic operational definitions, as advocated in the MISEV2023 guidelines [2]. Under this framework, investigators are expected to report the source material, separation workflow, and key biochemical features of the EV preparation, rather than relying on oversimplified subtype labels alone. Subsequent discussions around MISEV updates have further underscored that the field is still refining what should count as sufficient evidence for EV identity, comparability, and rigor [243]. Although this approach does not resolve heterogeneity, it provides a more realistic basis for reproducibility and regulatory translation.

Recognition of heterogeneity also complicates the concept of “purity” itself. In biofluids, EVs may not exist as isolated naked vesicles, but rather within a broader extracellular nanoparticle interactome and may acquire biomolecular coronas composed of proteins, nucleic acids, lipoproteins, and other circulating components [242, 391–394]. In some settings, these associations may influence apparent EV identity, biological behavior, and in vivo fate, suggesting that a simple contaminant-versus-purity framework may be insufficient. Rather than treating all associated material as analytically irrelevant, future work may need to consider vesicle–corona complexes more explicitly when interpreting EV function, product comparability, and therapeutic performance. This does not remove the need for rigorous purification, but it does support a more functionally informed view of what constitutes a clinically meaningful EV preparation [246].

## Engineering strategies for EV-based therapeutics

**Fig. 7:**
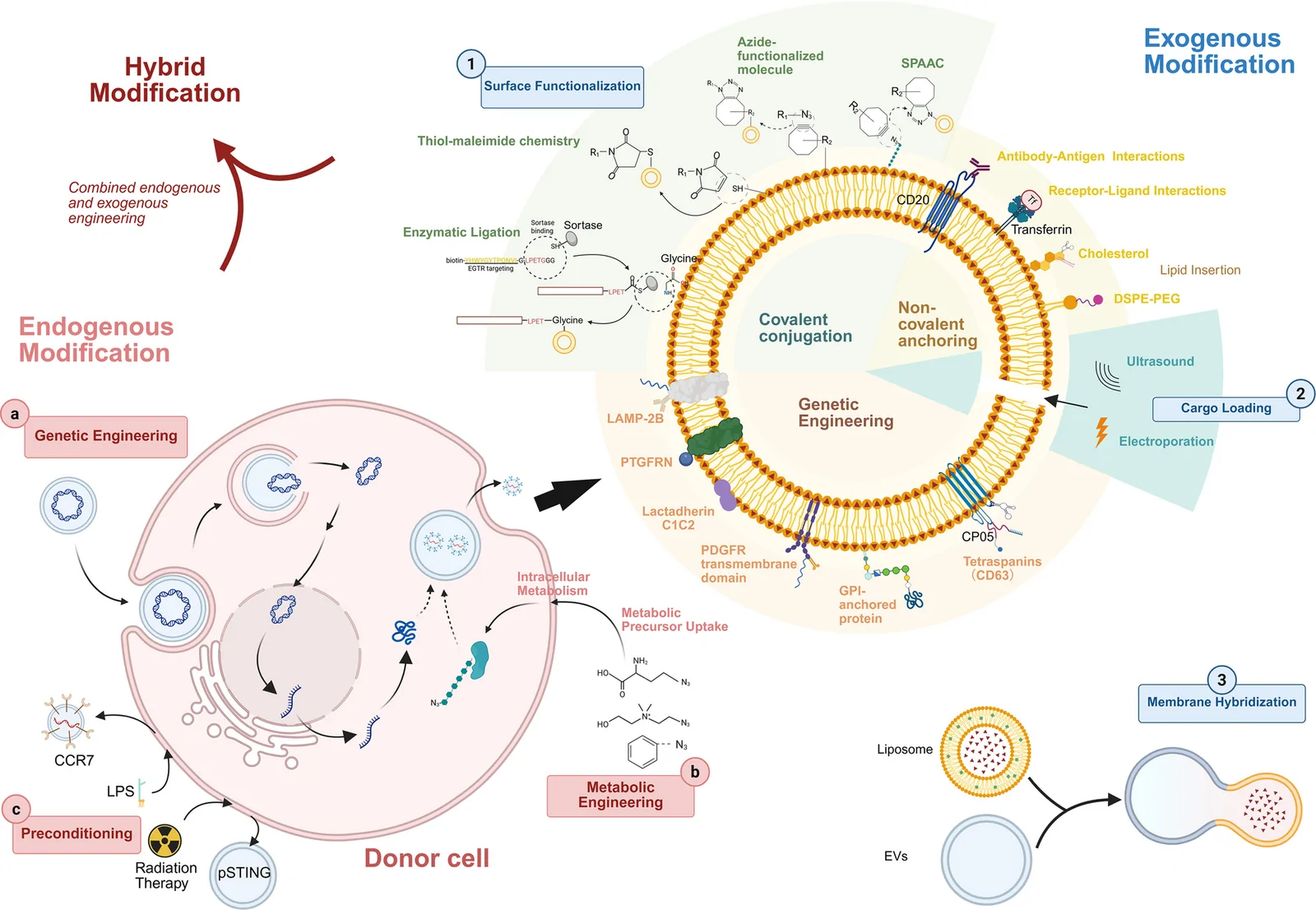
Major engineering strategies for the functional modification of EVs. Schematic overview of major engineering approaches used to enhance EV functionality for therapeutic delivery. Endogenous modification involves engineering donor cells to produce EVs with desired targeting, surface-display, or cargo-loading properties. Representative endogenous strategies include (a) genetic engineering, (b) metabolic engineering, and (c) preconditioning, which can alter EV composition, membrane features, or targeting behavior. Exogenous modification refers to post-isolation engineering of purified EVs and includes (1) surface functionalization, (2) cargo loading, and (3) membrane hybridization with synthetic or biological materials. Hybrid modification combines endogenous and exogenous strategies to generate EV products with combined targeting, loading, or surface-engineering features. Representative examples shown include membrane/scaffold protein fusion, bio-orthogonal metabolic labeling, covalent and non-covalent surface conjugation, passive or active cargo loading, and fusion with liposomal membranes. Abbreviations: EVs, extracellular vesicles; PEG, polyethylene glycol. Created with BioRender.com

Converting EVs from natural intercellular messengers into therapeutically useful delivery systems requires engineering strategies that improve cargo loading, targeting behavior, and in vivo performance while preserving product integrity and manufacturability. Current approaches generally rely on endogenous programming, exogenous modification, or hybrid combinations of the two. These methods increase engineering flexibility, but they also introduce additional challenges related to heterogeneity, stability, quality control, and translational reproducibility (Fig. 7).

### Endogenous and exogenous engineering

Current EV engineering strategies can be broadly divided into endogenous programming and exogenous modification, which differ in timing, controllability, and translational constraints. Endogenous engineering modifies the producer cell genetically or metabolically so that it secretes intrinsically altered EVs. This strategy can, in principle, link vesicle formation more closely to the intended engineered function and may be easier to align with controlled production workflows. However, it does not remove source- or process-dependent variability, and its greater developmental complexity may limit flexibility and prolong optimization.

Exogenous engineering, by contrast, modifies isolated EVs after purification through chemical or physical methods that alter the vesicle surface or load the lumen. This approach is often used because it allows relatively rapid testing and adaptation to different cargos or targeting requirements. At the same time, such flexibility may increase challenges related to in vivo stability, heterogeneity control, and release characterization. Neither endogenous nor exogenous engineering is therefore universally preferable. The appropriate strategy depends on the intended cargo, target tissue, required level of manufacturing control, and acceptable degree of product complexity [395]. A comparative overview of endogenous and exogenous engineering strategies is provided in Table 7.

**Table 7:** A comparative analysis of endogenous and exogenous EV engineering strategies

Feature	Endogenous Modification	Exogenous Modification
Core Principle	Modification of producer cells to generate producer-cell-programmed EVs using cellular biosynthetic machinery	In vitro chemical or physical alteration of pre-isolated EVs
Representative Methods	Genetic engineering (e.g., membrane protein fusion); Metabolic engineering (e.g., pre-installation of chemical handles); Cell pre-conditioning	Surface functionalization (e.g., click chemistry, lipid insertion); Cargo loading (e.g., electroporation, sonication); Membrane fusion/hybridization
Operational Complexity	High: Requires complex cell engineering, validation, and long development timelines	Low to Medium: Shorter development timelines and relatively straightforward procedures
Product Homogeneity	High: Functional molecules are homogeneously and often quantitatively integrated	Low to Variable: Modification sites and stoichiometry are often randomly distributed, leading to high heterogeneity
Modification Stability	High: Typically involves stable, covalent integration that is less prone to dissociation in vivo	Variable: Covalent modifications are stable; non-covalent modifications (e.g., lipid insertion) are at risk of dissociation in vivo
Impact on EV Bioactivity	Potential impact: Overexpression of foreign proteins may interfere with native EV biogenesis and function	Risk is method-dependent: Mild methods have minimal impact; harsh methods (e.g., electroporation) can severely compromise vesicle integrity
Versatility/Flexibility	Low: The strategy is often tied to a specific producer cell line and anchor protein, making it difficult to alter	High ("Plug-and-Play"): Highly versatile and can be flexibly applied to EVs from various sources for diverse modifications
Key Advantages	Homogeneous, stable, and reproducible product; well-suited for scalable and standardized manufacturing	Flexible, rapid, and universally applicable; well suited to rapid proof-of-concept testing and personalized modifications
Key Disadvantages	Long development timelines and high costs; safety and regulatory considerations for genetically modified cells	High product heterogeneity, posing challenges for quality control; modification process can damage EV integrity and function

"Endogenous modification" refers to the programming of producer cells via genetic or metabolic engineering prior to EV secretion. "Exogenous modification" involves the physicochemical manipulation of EVs after they have been isolated and purified from cell culture media or biofluids. Product Homogeneity assessment considers the consistency of cargo copy number per vesicle and the uniformity of surface ligand display across the EV population. "Plug-and-Play" refers to the modular capability of exogenous methods to functionalize a single batch of purified vesicles with various therapeutic agents

*Abbreviations*: *EVs* extracellular vesicles, *QC* quality control

Although conceptually distinct, these approaches are increasingly combined in hybrid designs. In such systems, endogenous programming can introduce defined reactive “handles” that are subsequently functionalized through exogenous conjugation. For example, metabolic engineering can incorporate azido-modified sugar precursors into EV surface glycans, thereby generating bio-orthogonal sites for click chemistry [396–400]. At a finer level, genetic-code expansion (GCE) enables site-specific incorporation of non-canonical amino acids bearing unique chemical groups into selected surface residues [401]. These hybrid strategies may improve control over surface modification or cargo engineering, but they also increase engineering and analytical complexity. At present, they are better viewed as enabling frameworks than as broadly mature solutions for clinical translation.

### Modifying tissue targeting

A major objective of EV engineering is to alter native biodistribution and improve delivery to selected tissues or cell populations. This is typically pursued by installing targeting ligands on the EV surface through endogenous or exogenous approaches [402]. However, the presence of a targeting ligand on the EV membrane does not by itself guarantee selective delivery in vivo, because ligand density, orientation, accessibility, stability, background organ tropism, and clearance behavior all remain important variables. More broadly, increased surface functionalization may improve targeting in selected models, but it does not resolve the broader difficulty of achieving predictable tissue selectivity under physiologically complex in vivo conditions [395].

#### Endogenous targeting strategies

Endogenous targeting strategies exploit cellular biosynthesis to generate intrinsically targeted vesicles by fusing a ligand, such as a peptide or nanobody, to an EV-enriched membrane protein that serves as an anchor. Canonical anchors such as Lamp2b [403–405] and tetraspanins including CD63 [406–408] were among the first systems used to demonstrate this approach, but their use can be limited by topology, cell-type dependency, and restricted tolerance for large or structurally complex ligands. These constraints have prompted exploration of alternative scaffolds with potentially greater structural tolerance. Examples include the transmembrane domain of PDGFR [409, 410], which can support display of more complex proteins such as bispecific antibodies, and PTGFRN, which has been explored as a scaffold for bulky payloads such as cytokines [411]. A comparative overview of representative protein anchors is provided in Table 8.

**Table 8:** A comparative overview of protein anchors for EV surface engineering

Anchor Protein/Platform	Displayed Ligand/Effector	Key Engineering Principle	Representative Application	Reference
Lamp2B	RVG peptide	Fusion to N-terminus of a native lysosomal protein	Brain-targeted delivery of siRNA across the blood–brain barrier	[403]
Lamp2B	Anti-HER2 affibody	Display of a small protein scaffold (affibody)	Co-delivery of chemotherapeutics and miRNA inhibitors to overcome drug resistance	[412]
Lamp2B	PSMA scFv	Display of a single-chain variable fragment (scFv)	Targeted delivery of a pro-pyroptotic protein (GSDMD) to prostate cancer	[413]
CD63	Apo-A1 peptide	Fusion to extracellular loop of a tetraspanin protein	Liver cancer targeting via scavenger receptor B1 for miR-26a delivery	[408]
CD81	Anti-HER2 fragment (trastuzumab light chain)	In continuous frame to full length tetraspanin	Targeted delivery of chemotherapeutics and long-chain mRNA to drug-resistant tumors	[414]
Syntenin-1	Anti-EGFR nanobody	Utilizing a scaffold protein involved in exosome biogenesis; fusion to its N-terminus	Generation of "universal immunomodulatory exosomes" for T-cell mediated tumor killing	[415]
GPI Anchor (from DAF)	Anti-EGFR Nanobody (EGa1)	Utilizing a Glycosylphosphatidylinositol (GPI) anchor signal peptide for membrane tethering; ligand highly enriched in EVs	Promoting EV binding to EGFR-expressing tumour cells under static and flow conditions	[416]
PDGFR-TM	GE11 peptide	Modular display using a generic transmembrane domain	EGFR-targeted delivery of tumor-suppressive miRNA to breast cancer	[417]
PDGFR-TM	αCD3/αEGFR bispecific scFv	Multi-chain fusion for complex functionality	T-cell redirection by bridging T cells and tumor cells ("SMART-Exos")	[409]
PDGFR-TM	SIRPα variant	Display of a native signaling protein for checkpoint blockade	Blocking the CD47 "don't eat me" signal to enhance phagocytosis	[418]
PTGFRN	IL12	Exploiting a large extracellular domain for bulky cargo	Localized immunotherapy with tumor-retained IL-12 for sustained immune activation	[419]
Lactadherin (C1C2 domain)	Tumor Antigens (PSA, PAP)	High-affinity binding to phosphatidylserine (PS) on the EV surface	Enhancing tumor vaccine immunogenicity by decorating EVs with antigens	[420]

Fusion protein engineering typically involves genetic modification of the producer cell to display ligands on either the N- or C-terminus of the anchor protein. Modular display refers to the use of synthetic or generic transmembrane domains that allow for the attachment of diverse effector molecules. Tetraspanins (e.g., CD63, CD81) are highly enriched in EV membranes and provide multiple loops for the insertion of targeting peptides or small protein scaffolds. References are indicated by numbers in parentheses corresponding to the main reference list

*Abbreviations*: *Lamp2B* lysosome-associated membrane protein 2B, *RVG* rabies virus glycoprotein, *siRNA* small interfering RNA, *HER2* human epidermal growth factor receptor 2, *PSMA* prostate-specific membrane antigen, *scFv* single-chain variable fragment, *GSDMD* gasdermin D, *Apo-A1* apolipoprotein A-I, *EGFR* epidermal growth factor receptor, *GPI* glycosylphosphatidylinositol, *DAF* decay-accelerating factor, *PDGFR-TM* platelet-derived growth factor receptor transmembrane domain, *SIRPα* signal regulatory protein alpha, *PTGFRN* prostaglandin F2 receptor negative regulator, *IL-12* interleukin-12, *PSA* prostate-specific antigen, *PAP* prostatic acid phosphatase, *PS* phosphatidylserine

Beyond static anchoring, some designs seek to engage endogenous trafficking machinery more directly. The LEAP platform, for example, incorporates an ESCRT-recognition motif to enhance multivalent antibody display on the EV surface by mimicking aspects of viral budding [421]. A more indirect endogenous strategy involves preconditioning producer cells—for example, lipopolysaccharide-stimulated dendritic cells—to upregulate surface molecules that may alter homing properties without direct genetic modification [422]. Endogenous targeting approaches may offer tighter integration with vesicle biogenesis, but their performance remains constrained by anchor topology, producer-cell context, and the need to preserve vesicle composition, stability, and manufacturability.

#### Exogenous targeting strategies

Exogenous targeting approaches allow EV surface properties to be modified after isolation. These strategies can be grouped broadly by their underlying chemical principle. Covalent conjugation seeks to establish stable linkages, with bio-orthogonal click chemistry among the most efficient methods currently available [423], particularly when upstream metabolic engineering is used to pre-install reactive groups on the EV surface [424, 425]. For applications requiring greater control over ligand orientation or site specificity, enzymatic or protein-based ligation systems such as Sortase A or SpyTag/SpyCatcher can be used, usually in combination with genetically introduced recognition tags [426–429].

Non-covalent approaches rely on milder biophysical interactions and are often preferred when membrane preservation is a priority. Representative methods include lipid insertion [425, 430–434], in which ligand–lipid conjugates are passively incorporated into the EV membrane, and adaptor-based systems that recognize endogenous EV surface molecules, such as the C1C2 domain of lactadherin for phosphatidylserine [420, 435, 436] or CP05 for CD63 [286, 437, 438]. More modular adaptor-based systems, including streptavidin–biotin strategies, further extend this modularity [439]. In selected cases, bio-affinity pairing has also been used to target specific EV subpopulations on the basis of unique surface markers, such as the transferrin receptor on reticulocyte-derived EVs [440] or CD20 on lymphocyte-derived EVs [441]. Exogenous targeting offers substantial design flexibility, but the resulting products may face challenges in ligand stability, steric shielding, batch-to-batch reproducibility, and release characterization. These issues are especially relevant for non-covalent modifications, in which premature dissociation in vivo remains a persistent concern [442].

### Modulating in vivo pharmacokinetics

Optimizing EV pharmacokinetics (PK) is important for improving delivery efficiency to target tissues. Strategies to reduce rapid clearance by the mononuclear phagocyte system (MPS) now include both passive shielding and more active modulation of host–vesicle interactions. The most established approach is chemical shielding, such as PEGylation or POxylation, which can mask the vesicle surface and reduce immune recognition [443]. A more biologically inspired strategy involves deliberate pre-formation of a controlled protein corona—for example with albumin—to modulate in vivo fate and immune interactions [394]. More recent approaches aim not only to modify the EV itself, but also to transiently modulate host clearance pathways. One example is the use of Sparstolonin B to inhibit macrophage TLR2 signaling and thereby prolong EV circulation and enhance tumor accumulation in selected models [444].

Together, these strategies indicate that EV pharmacokinetic engineering now includes both static surface shielding and more active modulation of host–vesicle interactions. However, PK optimization remains difficult to generalize, because interventions that prolong circulation or alter immune recognition may also affect biodistribution, comparability, and safety in product-specific ways. Pharmacokinetic engineering should therefore be interpreted not as a universally additive benefit, but as another design dimension requiring careful validation.

### Cargo loading strategies

The therapeutic performance of an EV product depends fundamentally on whether cargo can be introduced without compromising vesicle integrity. As with targeting, loading strategies can be divided into endogenous and exogenous approaches, each involving trade-offs among loading efficiency, controllability, and structural preservation.

#### Endogenous loading

Endogenous loading uses producer-cell machinery to package cargo during EV biogenesis. At the simplest level, passive enrichment can be achieved by increasing cytosolic cargo abundance, for example through overexpression of a protein or RNA of interest, or by exposing producer cells to a small-molecule drug to promote stochastic encapsulation [445–448]. Active endogenous loading, by contrast, introduces defined sorting signals to guide cargo into EVs more efficiently. Examples include embedding an RNA of interest within a pre-miR-451 backbone [449] or fusing proteins to loading-associated motifs such as sequences derived from ABI or BASP1 to hijack endogenous sorting pathways [411, 450, 451]. Non-genetic approaches, including radiotherapy or cytokine preconditioning, can also influence EV cargo composition by altering producer-cell state [452, 453].

These endogenous approaches may better preserve vesicle structure and may be more compatible with integrated manufacturing workflows, but they remain dependent on producer-cell biology and often provide only partial control over loading efficiency and batch-to-batch consistency.

#### Exogenous loading

Exogenous loading introduces cargo into purified EVs and is defined by a central tension between loading efficiency and vesicle preservation. Passive approaches, such as co-incubation with hydrophobic drugs or gentle transient permeabilization methods including osmotic shock, prioritize membrane integrity but generally show low and cargo-dependent efficiency [441, 454–457]. They may also promote surface adsorption or aggregation rather than true luminal encapsulation, thereby altering vesicle behavior and potentially increasing toxicity [442].

By contrast, active loading methods such as electroporation and sonication apply external energy to transiently permeabilize the membrane, increasing apparent loading efficiency for larger molecules such as nucleic acids [458, 459]. However, these approaches may compromise membrane structure, alter cargo distribution, or impair EV function [460]. Newer microfluidic platforms are being developed to apply more controlled forces at the single-vesicle level in an effort to improve loading while minimizing structural damage [461–464]. These approaches may improve control over loading and could support scalability, although preserving vesicle integrity, ensuring true luminal encapsulation, and maintaining product comparability remain major challenges.

### Hybrid, stimuli-responsive, and bio-inspired designs

Ecent EV engineering strategies increasingly combine targeting, cargo delivery, and environmental responsiveness within single products, but increased sophistication also introduces additional burdens in manufacturing, product definition, quality control, and regulatory evaluation. Greater design complexity should therefore not be equated with greater translational readiness, because gains in multifunctionality often introduce parallel challenges in scalability, reproducibility, and clinical standardization [395].

One route toward integration involves multifunctional chimeric peptides that combine targeting, therapeutic, and stimulus-responsive properties within a single molecular modification [465, 466]. Most of these systems, however, remain proof-of-concept constructs rather than mature translational platforms.

A related development is the construction of hybrid vesicles that combine natural and synthetic architectures. Fusion of EVs with liposomes is intended to combine the biological properties of EVs with the cargo capacity of synthetic nanocarriers. Although physical approaches such as extrusion can induce membrane fusion, more controlled bio-orthogonal strategies are also being explored [467–471]. One representative example uses DNA hybridization to mediate relatively gentle and specific membrane fusion, which may be advantageous for fragile cargos such as siRNA [472]. Such approaches may facilitate delivery of larger or more complex payloads, including in selected preclinical studies involving CRISPR/Cas9 systems, in selected preclinical settings [473]. Hybrid cellular systems, such as macrophage–tumor fusions, have also been explored as sources of EVs carrying features from both parent cells, although their translational role remains highly preliminary [474]. Yet each added layer of hybridization also increases analytical complexity and may complicate scale-up and regulatory definition.

Some EV designs have also incorporated synthetic-biology-inspired regulatory systems. Optogenetic systems, for example, have been incorporated into EV biogenesis pathways to enable light-triggered control over cargo loading or release. The EXPLORs system permits optical control of cargo incorporation [475], whereas the GIFTed-Exos platform has been used for site-specific light-induced release [476]. These systems allow experimentally precise control in preclinical settings, but their translational utility remains uncertain given the added complexity of light delivery, manufacturing, and in vivo validation.

To address lysosomal sequestration and limited cytosolic delivery, some groups have explored membrane-fusion-based strategies. By decorating EVs with fusogenic proteins such as VSV-G, “fusogenic EVs” may improve endosomal escape and enhance cytosolic delivery of bioactive proteins or nucleic acids in selected systems [450, 477, 478]. This strategy is particularly relevant for cargos that require cytosolic access, but added membrane engineering may further complicate product definition, safety assessment, and large-scale manufacturing. Many multifunctional and bio-inspired EV systems are better regarded as advanced preclinical design paradigms than as near-term translational standards.

## Therapeutic applications and clinical translation of engineered EVs

**Fig. 8:**
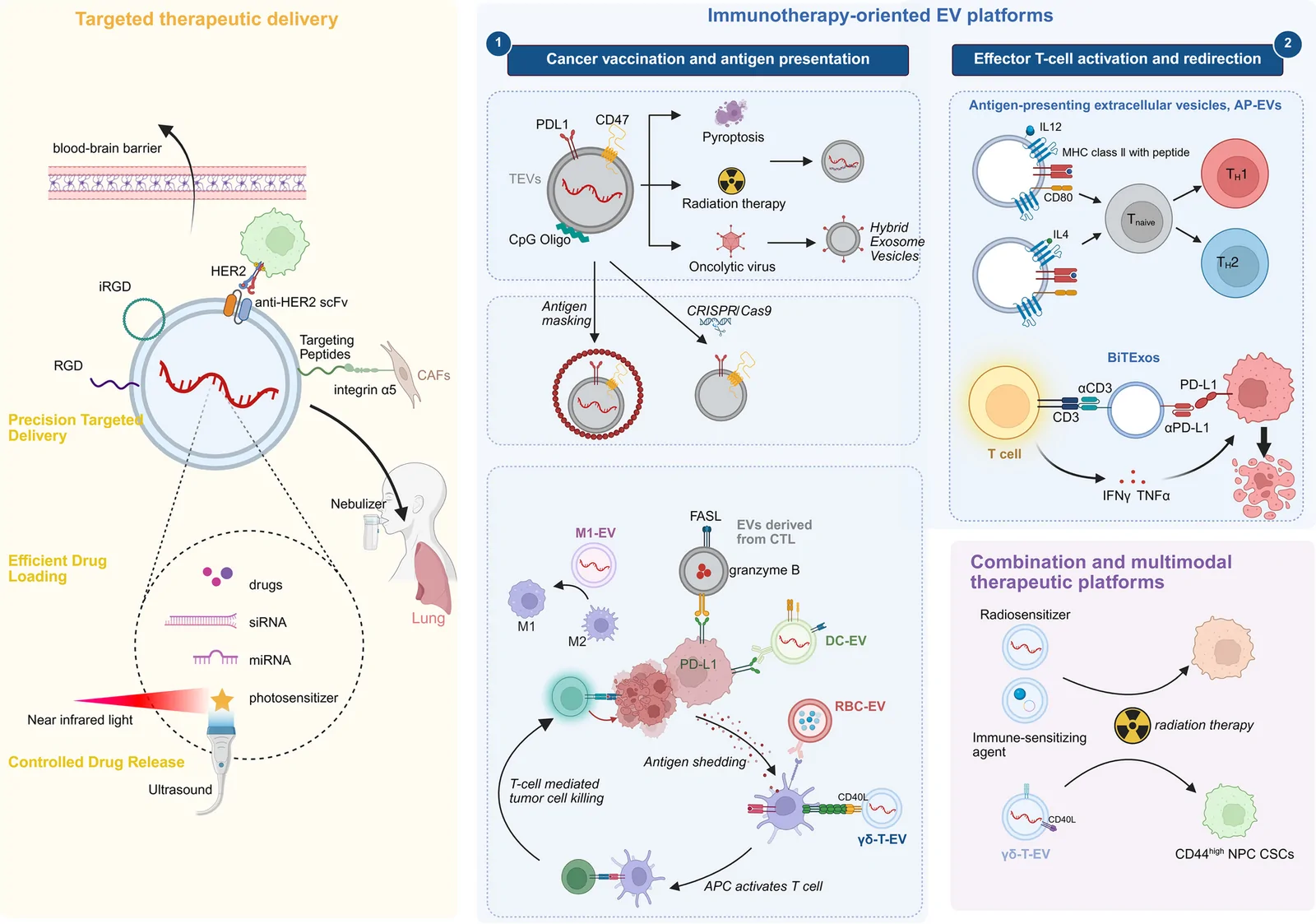
Major therapeutic application strategies of engineered EVs in oncology. Schematic overview of major therapeutic strategies using engineered EVs in precision oncology. (1) Targeted therapeutic delivery: EVs can be engineered to deliver chemotherapeutics, nucleic acids, proteins, and other payloads, with some systems incorporating stimulus-responsive release or tumor-microenvironment (TME) modulation. (2) Immunotherapy-oriented EV platforms: engineered EVs can potentiate anti-tumor immunity through two main routes: (a) cancer vaccination and antigen presentation, including adjuvant loading, immunogenic cell death (ICD)-associated vesicles, and APC-like EV platforms; and (b) effector T-cell activation and redirection, including artificial APC-like systems, co-stimulatory signaling support, and bispecific EV-based engagers that redirect cytotoxic T-cell activity. (3) Combination and multimodal therapeutic platforms: engineered EVs can be integrated with additional treatment modalities, including physical therapies, immune-sensitizing agents, or hybrid carrier systems, to support combined delivery, local sensitization, or coordinated immune modulation in selected settings. Abbreviations: EVs, extracellular vesicles; TME, tumor microenvironment; ICD, immunogenic cell death; APCs, antigen-presenting cells; DCs, dendritic cells; BiTExos, bispecific T-cell engagers. Created with BioRender.com

Engineered EVs are being explored as therapeutic platforms for several major challenges in solid tumor treatment. These applications can be grouped into three broad translational directions: drug delivery, modulation of antitumor immunity, and integration into multimodal therapeutic systems (Fig. 8). However, these directions differ substantially in maturity. Some are relatively closer to translational logic, whereas others remain largely proof-of-concept and continue to be constrained by manufacturing complexity, product heterogeneity, and incomplete clinical validation.

### Engineered EVs as drug delivery vehicles

Engineered EVs are increasingly being investigated as delivery vehicles for anticancer therapeutics because of their biocompatibility and their reported ability, in selected settings, to traverse biological barriers [479, 480]. A central goal of engineering is to overcome native limitations in cargo loading, targeting, and product consistency so that EVs can serve as more controllable delivery systems for diverse therapeutic payloads. These payloads include conventional chemotherapeutics, nucleic acid drugs directed against difficult targets such as KRAS [481–483], and innate immune agonists intended to sensitize tumors to immunotherapy [484–486] (Table 9).

**Table 9:** Representative engineering strategies for EV-based drug delivery

Source/Platform Type	Core Engineering & Payload	Therapeutic Strategy & Target	Translational Rationale	Ref
Normal Fibroblast	siRNA against oncogenic KRAS G12D	Silencing an "undruggable" driver mutation in pancreatic cancer (PDAC)	Landmark proof-of-concept for targeted RNAi therapy	[481]
NK Cell–Liposome Hybrid	Ferroptosis inducer (RSL3)	Hybrid vesicle platform combining EV-mediated targeting with liposomal cargo capacity for glioma	Synergistic ferroptosis-immunotherapy; Advanced platform design	[487]
BMSC	Anti-fibrotic agents; Surface-targeted to CAFs	Systemic delivery of drugs to remodel the tumor stroma in pancreatic cancer	Targeting the TME instead of cancer cells	[488]
Cancer Stem Cell (CSC)	Ginsenoside Rb1; Surface-targeted to PI3K-mutant NSCLC	Delivery of a natural product to overcome therapy resistance in cancer stem cells	Targeting the CSC niche and modulating resistance pathways	[489]
Autologous Tumor Cell	STING agonist (cGAMP)	Leveraging homotypic targeting for in situ activation of innate immunity in melanoma	Combining tumor-homing delivery with potent immunotherapy	[484]
Red Blood Cell (RBC)	KRAS ASO and RIG-I agonist (immRNA)	Using a safe, scalable chassis for co-delivery of antisense oligonucleotides and immunomodulators	"Off-the-shelf" platform for combined gene silencing and immune activation	[485]
M1 Macrophage	Hypoxia-activated prodrug + Photosensitizers	Exploiting natural cell tropism for multi-modal therapy of glioblastoma	Overcoming the blood–brain barrier; Stimulus-responsive drug release	(490)

"Undruggable" targets refer to oncogenic drivers (e.g., KRAS G12D) that have historically been difficult to target with conventional small-molecule inhibitors. Homotypic targeting exploits the innate tendency of TEVs to home back to their cells of origin. References are cited as they appear in the main reference list

*Abbreviations*: *EV* extracellular vesicle, *BMSC* bone marrow-derived mesenchymal stem cell, *CSC* cancer stem cell, *RBC* red blood cell, *PDAC* pancreatic ductal adenocarcinoma, *NSCLC* non-small cell lung cancer. *TME* tumor microenvironment, *KRAS* Kirsten rat sarcoma virus, *ASO* antisense oligonucleotide, *RNAi* RNA interference, *STING* stimulator of interferon genes

Beyond simple cargo delivery, EV-based systems are also being explored to address specific therapeutic barriers, such as fibrotic stromal restriction [488], blood–brain barrier access in selected macrophage-EV systems [490], or localized administration routes such as nebulized inhalation for mRNA delivery to lung tumors [491, 492]. Stimulus-responsive and theranostic EV platforms that exploit pH, hypoxia, or other tumor-associated cues are also being explored [493–495]. However, most such systems remain preclinical, and improved performance in experimental models does not necessarily translate into predictable clinical advantage, particularly when cargo loading, targeting behavior, and large-scale product consistency remain incompletely resolved. EV-based drug delivery is therefore best regarded as one of the more translationally plausible directions in the field, albeit one that still faces substantial product-development and validation challenges.

### Engineered EVs in antitumor immunotherapy

In cancer immunotherapy, engineered EVs are being explored as systems that may influence different stages of the cancer–immunity cycle. Depending on design, they may support antigen presentation, enhance effector-cell activity, or influence suppressive tumor microenvironments, although most remain at the stage of advanced preclinical development.

#### Cancer vaccines

Engineered EVs offer a cell-free format for antigen and adjuvant delivery to antigen-presenting cells (APCs). One focus has been optimization of the antigen-presentation platform itself. Native TEVs can provide access to personalized antigen repertoires but are often weakly immunogenic or even immunosuppressive [496, 497]. Two broad strategies have emerged. One seeks to “renovate” TEVs by enhancing immunogenicity through adjuvants or damage-associated molecular patterns (DAMPs) [152, 498–501], or by removing or masking inhibitory signals such as CD47 [112, 502, 503]. The other replaces the source entirely, using EVs derived from professional APCs such as dendritic cells, which may provide a more intrinsically immunogenic vaccine platform [504–506].

A second direction combines antigen presentation with checkpoint-related or TME-modulating components in an effort to improve vaccine performance [399, 507–510]. A third, conceptually distinct direction is in situ autovaccination. Rather than delivering predetermined antigens, these systems attempt to mobilize endogenous APCs within tumors so that the tumor itself becomes a source of immunogenic antigen release. By inducing immunogenic cell death (ICD) or recruiting APCs through local chemokine signals, such strategies may generate individualized immune responses based on the patient’s native neoantigen landscape [511–513]. Although this approach may better capture patient-specific antigenic diversity, the extent to which it can be standardized, individualized, and clinically scaled remains uncertain.

#### Enhancing T-cell effector function

A major challenge in immunotherapy is sustaining cytotoxic T-cell activity within a suppressive tumor environment. Engineered EVs are being investigated to address this problem through two broad strategies: artificial antigen-presenting cell-like systems and bispecific T-cell engager-like platforms.

The artificial APC strategy seeks to recreate the core signals required for T-cell activation by displaying peptide–MHC complexes, co-stimulatory ligands such as CD80/CD86, and, in some cases, cytokine-associated signals on the EV surface. Such antigen-presenting EVs have been reported to prime and expand tumor-specific T cells in preclinical models [514, 515]. These platforms can also be modified to include checkpoint-related or additional co-stimulatory modules, although such additions further complicate product definition and potency assessment [516, 517]. A related but mechanistically distinct strategy is the development of bispecific T-cell-targeting EVs (BiTExos), which co-display binding moieties for tumor-associated antigens and CD3 on T cells, thereby enforcing proximity and promoting redirected cytotoxicity [421]. Integrated systems such as GEMINI-Exos extend this principle by combining T-cell redirection, checkpoint-related signaling, and co-stimulation within a single vesicle [410].

However, increasing the number of engineered immune functions within one EV product may also complicate vesicle characterization, potency assessment, in vivo persistence, and regulatory classification. Such systems may help enhance T-cell function in preclinical settings, but many of the most sophisticated designs should still be regarded as proof-of-concept systems rather than near-term translational standards.

#### Remodeling the immunosuppressive microenvironment

Even when immune priming and effector activation are achieved, responses may still be attenuated by the immunosuppressive TME. Accordingly, direct modulation of the TME has emerged as a complementary engineering objective. Engineered EVs are being explored both to eliminate suppressive stromal or myeloid components and to reprogram immune-cell states within tumors. One approach targets specific suppressive populations directly. Examples include EVs with myeloid tropism delivering agents such as CSF-1R inhibitors to deplete tumor-associated macrophages (TAMs) or myeloid-derived suppressor cells (MDSCs) [518], as well as M1 macrophage-derived EVs designed to re-educate M2-like macrophages toward a more pro-inflammatory state [519]. More aggressive strategies have also been explored, such as EVs displaying DR5 agonist antibodies to induce apoptosis across multiple suppressive compartments, including tumor cells, CAFs, and MDSCs [520].

Another strategy is to reshape local immune organization rather than simply deplete suppressive populations. In one preclinical study, engineered EVs incorporating activated STING, an anti-CD47 nanobody, and an autophagy inhibitor were reported to induce tertiary lymphoid structure (TLS)-like formation in glioma models [453]. Such findings indicate that engineered EVs may, in selected settings, influence local immune organization in ways that could support more sustained antitumor responses. Whether this type of architectural immune remodeling can be generalized across tumor types, standardized across products, or integrated into clinically tractable EV platforms remains unknown.

### Multimodal therapeutic combinations

As tumor complexity and therapeutic resistance continue to limit monotherapies, engineered EVs are being explored as carriers for multimodal treatment strategies that combine molecular payload delivery with physical or biologically active partner interventions. While this concept is attractive, increasing multimodal complexity also makes mechanistic attribution, manufacturing control, and regulatory evaluation more difficult.

Engineered EVs have shown potential in combination with physical therapies such as radiotherapy and phototherapy. As targeted carriers, they can deliver radiosensitizers or photoactive agents to tumor sites and may thereby improve local treatment efficiency while potentially reducing off-target toxicity [521, 522]. EVs can also be used to co-deliver immune-stimulatory molecules such as STING agonists during local tumor ablation, thereby attempting to convert local damage into a more systemic antitumor immune response [523, 524]. However, the therapeutic contribution of each component in these multimodal systems can be difficult to disentangle, and integration of multiple functional layers may exceed what is currently feasible for standardized translation.

A more complex example is the combination of engineered EVs with live biotherapeutics. In one preclinical study, an engineered probiotic secreting IL-32 was combined with EV-associated antioxidant selenium nanoparticles for oral administration in colorectal cancer models, with the aim of enhancing CD8⁺ T-cell function while reducing oxidative stress in the TME [525]. This type of system combines microbial engineering, nanodrug design, and EV-based delivery within a single preclinical construct. At the same time, such highly composite strategies are translationally demanding, and their manufacturability, comparability, and regulatory pathway are likely to be substantially more complex than those of simpler EV products.

### Lessons from early clinical trials

Early clinical studies of EV-based cancer therapeutics have mainly involved cancer vaccine approaches and EV-mediated delivery strategies. Although these studies were limited in scale and mechanistic resolution, they have provided important lessons regarding both the promise and the constraints of EV-based therapy (Table 10). The most informative experience to date has come from early trials using relatively natural EV formulations, particularly dendritic cell-derived exosomes (Dex) and ascites- or tumor-derived EVs as cancer vaccines.

**Table 10:** Representative Clinical Trials of EV-Based Interventions in Oncology

Disease Indication	Phase	Therapeutic Agent/Intervention	EV Source	Strategy	Clinical Status/Reported Findings	Registration Number	Reference
Colorectal Cancer	I	Autologous Ascites-derived Exosomes (Aex) ± GM-CSF	Tumor Ascites	Cancer Vaccine	Safe; Combination with GM-CSF required for CTL response	/	[526]
Melanoma	I	Autologous MAGE-3 peptide-pulsed DC-EVs (Dex)	DC	Cancer Vaccine	Safe; Limited T-cell response; Some clinical responses noted	/	[527]
NSCLC	I	Autologous MAGE peptide-loaded DC-EVs (DEX)	DC	Cancer Vaccine	Safe; Minimal T-cell response but increased NK cell activity	/	[528]
NSCLC (Advanced, Maintenance)	II	Autologous MAGE-loaded Dex (IFN-γ matured)	DC	Cancer Vaccine	Missed primary PFS endpoint; NK cell function strongly correlated with survival	NCT01159288	[529]
Bladder Cancer	I	Chimeric exosomal tumor vaccine	Autologous Tumor-APC Hybrids	Personalized Cancer Vaccine	Recruiting	NCT05559177	/
Pancreatic Cancer (KRAS G12D)	I	Allogeneic MSC-derived exosomes loaded with KRASG12D siRNA (iExosomes)	MSC	Targeted Drug Delivery/RNAi Therapy	Recruiting	NCT03608631	[530]
HCC & Liver Metastases (Gastric/CRC)	I	Engineered exosomes loaded with STAT6 ASO (exoASO-STAT6/CDK-004)	Engineered Human Cell Line	TME Immunomodulation/RNAi Therapy	Terminated (due to company bankruptcy)	NCT05375604	/
Lung Cancer with Malignant Pleural Effusion (MPE)	I	Intrapleural autologous tumor-derived MPs packaging methotrexate (ATMPs-MTX)	Autologous Tumor Cells	Targeted Drug Delivery/Immunomodulation	Safe & high ORR in MPE; Induced anti-tumor T-cell responses	NCT02657460	[531]
Colon Cancer	I	Oral Plant exosome-conjugated Curcumin vs. Curcumin	Plant	Oral Drug Delivery/Bioavailability Enhancement	Recruiting	NCT01294072	/
Chemotherapy-induced Myelosuppression (in AML)	I	Allogeneic Umbilical Cord MSC-derived exosomes (UCMSC-Exo)	Umbilical Cord MSCs	Regenerative/Supportive	Recruiting	NCT06245746	/
Oral Mucositis (in Head & Neck Cancer)	I	Oral Grape-derived exosomes vs. Standard of Care	Grape	Ameliorate Toxicity	Completed. Results not posted	NCT01668849	/

Registration Numbers refer to identifier codes from ClinicalTrials.gov (NCT numbers). "Terminated" status for NCT05375604 (Codiak BioSciences) highlights the significant financial and translational barriers facing the EV industry, despite promising preclinical data. Phase represents the clinical development stage (I: Safety/Dose-finding; II: Preliminary efficacy)

*Abbreviations*: *EVs* extracellular vesicles, *Aex* ascites-derived exosomes, *Dex* dendritic cell-derived exosomes, *MSC* mesenchymal stem cell, *NSCLC* non-small cell lung cancer, *HCC* hepatocellular carcinoma, *CRC* colorectal cancer, *AML* acute myeloid leukemia, *MPE* malignant pleural effusion, *CTL* cytotoxic T lymphocyte, *NK* natural killer, *PFS* progression-free survival, *ORR* objective response rate; *ASO* antisense oligonucleotide, *GM-CSF* granulocyte–macrophage colony-stimulating factor

Across multiple early-phase studies, these formulations generally showed favorable safety and tolerability, supporting the overall biocompatibility of EV-based interventions. However, clinical efficacy was modest, and robust tumor-specific adaptive T-cell responses were seldom observed [528]. In several studies, the more consistent immunological signal was enhancement of innate immune activity, particularly NK-cell activation. In some cases, prolonged progression-free survival correlated more closely with increased NKp30-related NK activity than with detectable expansion of cytotoxic T cells [527, 529]. Similarly, in a colorectal cancer study, ascites-derived EVs alone failed to induce meaningful CEA-specific CTL responses, whereas co-administration with GM-CSF improved immunogenicity and promoted antigen-specific T-cell activation [526]. These findings suggest that the immune effects of early EV vaccine platforms may have been more dependent on innate or adjuvant-like mechanisms than originally anticipated.

These clinical data point to several constraints that likely limited the potency of first-generation EV vaccines. At the formulation level, peptide pulsing and related antigen-loading strategies were inefficient, whereas natural EV preparations could also contain inhibitory cargoes such as TGF-β. At the pharmacokinetic level, subcutaneous administration likely restricted lymphatic trafficking and limited effective interaction with APCs. At the trial-design level, endpoint selection often prioritized adaptive T-cell readouts while underestimating the possible importance of innate immune activation. Retrospective analyses suggest that NK-cell activation, rather than conventional T-cell priming, may have been a more informative correlate of benefit in some Dex studies [532], consistent with the ability of these vesicles to engage NK cells through ligands such as NKG2D ligands and IL-15Rα.

These studies suggest that the main limitations of first-generation EV vaccines were not safety, but inadequate antigen loading, incomplete control of inhibitory cargo, and insufficient attention to trafficking and immune endpoints. More defined antigen-loading strategies, better control of inhibitory cargo, and more selective immune-engineering designs may be required to improve consistency and potency. Pharmacokinetic optimization—through improved trafficking, sustained-release approaches, or better control of lymphoid exposure—also appears likely to be important. Future clinical studies will likely need biomarker-guided designs and broader endpoint selection that includes innate immune activation and microenvironmental remodeling alongside T-cell-centered readouts. Early clinical experience has therefore not yet validated EVs as broadly effective cancer immunotherapies, but it has clarified where the next generation of engineered EV products may need to improve.

## Challenges in the clinical translation of engineered EVs

Despite growing interest in EV-based therapeutics, substantial barriers remain before these systems can become broadly viable clinical and commercial products. Scientific complexity, manufacturing constraints, and insufficient regulatory frameworks remain major obstacles to clinical translation of engineered EVs [326]. Optimism regarding engineered EV therapeutics should therefore be tempered by a realistic appraisal of the major barriers that still limit clinical development.

### Standardization, purity, and product definition

A central barrier to clinical translation is the difficulty of isolating EV preparations with sufficiently well-defined identity and purity. Because currently available separation methods often struggle to balance yield and purity, final products may remain contaminated by lipoproteins and non-vesicular protein complexes that overlap with EVs in size and density. In practice, relatively few studies fully satisfy experimental standards sufficient to support strong claims regarding EV purity and identity [533]. Additional concern arises from limitations in tracking methodology. Widely used lipophilic dyes such as PKH26 can form non-specific aggregates in vivo, raising the possibility that some reported “EV biodistribution” patterns may partly reflect labeling artifacts rather than authentic vesicle behavior [534]. Moreover, EV surfaces readily adsorb circulating proteins and may acquire complex protein coronas [535]. These issues complicate both product definition and the assignment of critical quality attributes (CQAs).

Some newer methods may improve specific aspects of separation or throughput, but none yet resolves the broader standardization problem. Zwitterionic coacervate-based phase-separation strategies have been proposed as approaches to improve EV–lipoprotein separation in complex fluids [536], whereas automated microplate-based SEC platforms may help alleviate throughput limitations [537]. More broadly, progress will depend on stricter hierarchical characterization workflows and wider use of single-vesicle analytical approaches to distinguish true luminal encapsulation from non-specific surface association and to define product identity more rigorously [538, 539].

### CMC bottlenecks and manufacturing scale-up

CMC challenges become substantially more demanding once EV products move from laboratory-scale studies toward clinical development. Stable, large-scale production under GMP-compatible conditions remains difficult. Upstream, robust producer-cell systems are needed that can maintain acceptable yield and product consistency across extended culture periods [540–542]. Downstream, purification processes must remove nanoscale contaminants without causing excessive vesicle loss or damage [543–545]. One particularly important issue is batch-to-batch variation: relatively small changes in the physiological state of producer cells can significantly alter EV cargo composition [546]. At present, the absence of standardized release criteria and the lack of EV-specific regulatory guidance from agencies such as the FDA or EMA remain major constraints on product approval and broader clinical development.

Bioprocess engineering is beginning to address parts of this problem. Upstream, the field is increasingly exploring 3D perfusion-based systems rather than conventional 2D culture, and both hollow-fiber bioreactors (HFBs) and stirred-tank bioreactors (STRs) have been investigated as platforms for increasing production scale [547]. Source selection is also critical. Carefully characterized engineered producer lines, such as suspension-adapted HEK293F cells, have been reported to support relatively stable molecular and physicochemical product attributes across passages and production runs under selected conditions [548]. Even so, scalable manufacturing, rigorous characterization, and product comparability remain prerequisites for any credible regulatory path for engineered EV therapeutics [549].

### Pharmacokinetics and mononuclear phagocyte clearance

Although engineered EVs are being explored as targeted delivery vehicles, their pharmacokinetic behavior remains a major barrier to systemic administration. After intravenous injection, unmodified EVs often display circulation half-lives measured in minutes, with a large fraction rapidly sequestered by the liver—particularly Kupffer cells—and the spleen.

Recent studies suggest that the dynamic protein corona acquired after entry into the bloodstream is an important determinant of EV clearance kinetics. Naturally formed coronas may facilitate non-specific recognition and uptake by monocytes and macrophages [394]. In vivo tracking studies further indicate that, even under pathological conditions such as systemic inflammation, corona remodeling may increase EV accumulation and uptake in selected organs while still producing very limited functional cytosolic delivery in target cells [535]. These findings underscore that biodistribution alone should not be equated with effective therapeutic delivery. Several surface-engineering strategies are being explored to reduce premature immune clearance. For example, deliberate modulation of the corona through albumin precoating has been reported to reduce non-specific hepatic macrophage uptake and increase distribution toward parenchymal target cells [394]. Biomimetic “don’t eat me” strategies have also been investigated. For example, chimeric CD47–CD63 display on EV membranes has been shown to engage SIRPα and prolong circulation in preclinical settings [550]. Reducing corona-mediated immune recognition is likely to be necessary for improving systemic EV delivery efficiency, although more favorable biodistribution should still not be equated with effective intracellular delivery.

### Heterogeneity and the biomarker-to-targeting mismatch

#### Multiscale heterogeneity as a translational barrier

Among the biological hurdles to translation, heterogeneity remains one of the most difficult to control. It operates across spatial, cellular, and molecular scales. At the tissue level, tumors are not only structurally heterogeneous but also functionally compartmentalized. In HER2-positive breast cancer, for example, clinical imaging has identified tumor regions enriched in PD-L1^high EV-secreting cells, forming localized immune-evasive niches that are relatively shielded from T-cell infiltration [551]. At the cellular level, functionally distinct EV populations may be released not only by different molecular subtypes, such as triple-negative breast cancer versus other breast cancer categories [552], but also by rare and aggressive subpopulations within the same tumor [551]. At the single-vesicle level, this heterogeneity persists further: a population defined macroscopically as “HER2-positive” may still contain a substantial fraction of vesicles with low or undetectable HER2 expression (363). This creates a dynamic and heterogeneous target profile that limits the reliability of single-marker targeting strategies.

#### Dynamic target expression and context-dependent target biology

Targeted design is further complicated by the instability and contextual fluidity of molecular information. A mismatch may exist between the molecular profile inferred from circulating biomarkers, including EV-associated signals, and the real-time biological state of the tumor. Target expression is not static, but can be altered by therapy and by the microenvironment, meaning that a strategy designed around a single time-point measurement may fail as tumor biology evolves [553]. In addition, the biological function of some targets is itself context-dependent. For example, MEK1 may be tumor-promoting in one setting yet exert different effects when transferred within EVs [214]. Likewise, molecules such as PD-1 can exist in membrane-bound and EV-associated forms with potentially distinct biological roles [554]. Targeting such molecules without sufficient contextual understanding may therefore produce unintended biological effects. This mismatch underscores a major translational limitation: biomarker-derived targeting assumptions may not remain valid as tumor biology evolves, and practical approaches to address this remain unresolved.

### Safety, dose, delivery, and the therapeutic window

Engineered EVs must also overcome fundamental pharmacological constraints, because their biological effects depend not only on intrinsic design, but also on how they are administered. The cell of origin influences innate biodistribution, whereas extrinsic variables—including route of administration and dose—substantially shape the in vivo response. Systemic versus local delivery can produce markedly different tissue-distribution patterns, and dose may alter not only efficacy but the nature of the biological response itself [541, 555–557].

Dose dependence is particularly important and remains insufficiently standardized across the field. High, non-physiological doses commonly used in vitro can trigger non-specific cellular responses, such as lysosomal activation, that obscure the intended mechanism of action and may partly explain some in vitro–in vivo translation failures. In vivo, rapid clearance by the MPS further limits effective delivery [541]. Safety concerns also remain substantial and include potential immunogenicity of engineered components [517], off-target toxicity of the therapeutic payload, and, for certain EV sources, the theoretical risk of horizontal transfer of oncogenic material [558, 559]. Balancing efficacy and biosafety will therefore require clearer definition of the therapeutic window, supported by pharmacokinetic modeling, biodistribution analysis, dose–response studies, and longer-term safety evaluation.

## Conclusion and outlook

Progress in EV research has reshaped how solid-tumor biology and EV-based therapeutics are understood. EVs are no longer viewed simply as mediators of intercellular communication, but as context-dependent systems through which tumor behavior, immune remodeling, metastasis, and therapeutic adaptation can be examined. At the same time, the same properties that make EVs biologically informative—including heterogeneity, membrane plasticity, and context-dependent cargo sorting—also make them difficult to interpret and challenging to translate.

A central lesson from this field is that progress in engineered EVs depends less on abstract design alone than on increasingly detailed understanding of EV biology itself. Advances in intracellular trafficking, lipid metabolism, source selection, cargo loading, targeting, and product definition have expanded the scope of EV engineering, but they have also clarified how many of the key barriers are biological as well as technical. EV heterogeneity, incomplete control of cargo composition, limited pharmacokinetic predictability, manufacturing variability, and unresolved regulatory standards remain major constraints on translation.

For this reason, the future impact of engineered EVs will depend less on the conceptual appeal of increasingly complex designs than on whether they can be made more reproducible, better defined, and more rigorously validated. At present, many of the most sophisticated systems remain advanced preclinical constructs rather than near-term therapeutic products. The key translational challenge is therefore not simply to engineer additional functionality, but to determine which EV properties are biologically meaningful, therapeutically useful, and compatible with scalable manufacturing and acceptable safety.

Overall, engineered EVs should be regarded as a promising but still emerging therapeutic modality. They are unlikely to replace existing treatment platforms, but they may become useful additions to the therapeutic landscape if current barriers in standardization, manufacturing, pharmacology, safety evaluation, and clinical validation can be reduced. A more realistic near-term goal is therefore not broad clinical transformation, but the development of clearly defined EV products that can demonstrate reproducible value in selected applications within precision oncology.

## Data Availability

No datasets were generated or analysed during the current study.

## Abbreviations

AF4: Asymmetric flow field-flow fractionation
APCs: Antigen-presenting cells
ASO: Antisense oligonucleotide
BBB: Blood–brain barrier
bEVs: Bacterial extracellular vesicles
BiTExos: Bispecific T-cell-engaging extracellular vesicles
CAFs: Cancer-associated fibroblasts
CAR: Chimeric antigen receptor
CLIA: Clinical Laboratory Improvement Amendments
CMC: Chemistry, manufacturing, and controls
CNS: Central nervous system
CQAs: Critical quality attributes
CTCs: Circulating tumor cells
CTLs: Cytotoxic T lymphocytes
DAMPs: Damage-associated molecular patterns
DCs: Dendritic cells
DGC: Density gradient centrifugation
dUC: Differential ultracentrifugation
EE: Early endosome
ESCRT: Endosomal sorting complex required for transport
EVs: Extracellular vesicles
FDA: U.S. Food and Drug Administration
GMP: Good manufacturing practice
HCC: Hepatocellular carcinoma
HFB: Hollow-fiber bioreactor
ICD: Immunogenic cell death
ICIs: Immune checkpoint inhibitors
ILVs: Intraluminal vesicles
ISEV: International Society for Extracellular Vesicles
LDT: Laboratory developed test
lncRNA: Long non-coding RNA
LPS: Lipopolysaccharide
miRNA: MicroRNA
MSCs: Mesenchymal stem cells
MVBs: Multivesicular bodies
NETs: Neutrophil extracellular traps
nFCM: Nano-flow cytometry
NK: Natural killer
NMPA: National Medical Products Administration
NSCLC: Non-small cell lung cancer
PDAC: Pancreatic ductal adenocarcinoma
PDEVs: Plant-derived extracellular vesicles
PD-1: Programmed cell death protein 1
PD-L1: Programmed cell death-ligand 1
PMN: Pre-metastatic niche
RE: Recycling endosome
sEVs: Small extracellular vesicles
SEC: Size-exclusion chromatography
SIRPα: Signal regulatory protein alpha
STR: Stirred-tank bioreactor
TAMs: Tumor-associated macrophages
TANs: Tumor-associated neutrophils
TEVs: Tumor-derived extracellular vesicles
TFF: Tangential flow filtration
TGF-β: Transforming growth factor beta
TLS: Tertiary lymphoid structures
TME: Tumor microenvironment
Tregs: Regulatory T cells
